# Comprehensive Analysis of Bias in TEMPO NO_2_ Column Densities Through Pandora Observations

**DOI:** 10.1029/2025JD044150

**Published:** 2025-08-16

**Authors:** Masoud Ghahremanloo, Caroline Nowlan, Gonzalo González Abad, Cecilia Garraffo, Xiong Liu, Huiqun Wang, Barron Henderson, Eric Baumann, Lukas Valin, Jeffrey A. Geddes, Xiaoyi Zhao

**Affiliations:** 1 Center for Astrophysics | Harvard & Smithsonian, Cambridge, MA, USA; 2 Center for Environmental Measurements and Modeling, Office of Research and Development, U.S. Environmental Protection Agency, Research Triangle Park, NC, USA; 3 Department of Earth & Environment, Boston University, Boston, MA, USA; 4 Air Quality Research Division, Environment and Climate Change Canada, Toronto, ON, Canada

## Abstract

Tropospheric Emissions: Monitoring of Pollution (TEMPO) is the first geostationary satellite instrument to monitor air pollutants across North America. This study uses Pandora observations to analyze the bias in TEMPO Level-3 total column density of NO_2_ (TOTNO_2_) from August 2023 to December 2024. TEMPO achieves high accuracy at 5% cloud-filtering threshold: correlation coefficient (R) of 0.86, index of agreement (IOA) of 0.91, mean absolute bias (MAB) of 1.423 × 10^15^ molecules/cm^2^, and a percentage MAB (MABP) of 23.1%, corresponding to a 12% underestimation. Accuracy decreases when pixels with greater cloud-cover are included. Solar zenith angle (SZA) of 10–20° yields the highest accuracy (R: 0.87, MABP: 22.7%), whereas SZAs of 70–80° yield the lowest (R: 0.71, MABP: 35.2%). Consequently, early-morning or near-sunset observations are less reliable than midday. This discrepancy could stem from inaccurate simulation of diurnal variations in the boundary-layer height in the a-priori, and from larger uncertainties in radiative transfer at high SZAs. TEMPO overestimates TOTNO_2_ at low NO_2_ levels and underestimates at high levels, with maximum biases of +16% (low) and − 31% (high), respectively. Station-to-station performance varies considerably, with R ranging from 0.29 to 0.84 and MABP from 14.9% to 49.3%. Stations situated at higher altitudes relative to the ground show reduced agreement with TEMPO, as Pandora cannot detect NO_2_ below the instrument’s altitude, whereas TEMPO retrieves the full column. Validation of TEMPO TOTNO_2_ at TROPOMI overpass time indicates that TEMPO’s performance relative to Pandora (IOA: 0.93, MABP: 22.3%) closely matches that of TROPOMI (IOA: 0.92, MABP: 20.1%).

## Plain Language Summary

Several satellite instruments measure trace gases in the Earth’s atmosphere. These instruments operate either in sun-synchronous orbits, rotating the Earth and covering different regions sequentially, or in geostationary orbits, remaining fixed over a specific location on Earth. The Tropospheric Emissions: Monitoring of Pollution (TEMPO), launched in April 2023, is the first geostationary satellite instrument to measure criteria air pollutants hourly over North America. One pollutant monitored by TEMPO is nitrogen dioxide (NO_2_), known for its harmful effects on human health and the environment. Continuous hourly monitoring of NO_2_ at high spatial resolution via TEMPO significantly helps scientists and policymakers in controlling NO_2_ levels and identifying emission sources in North America. This study validates TEMPO’s total column density of NO_2_ (TOTNO_2_) using observations from Pandora instruments. It further analyzes factors influencing TEMPO TOTNO_2_ accuracy, providing scientists and users with a better understanding of its reliability under various conditions. Results demonstrate TEMPO’s high accuracy, indicated by a correlation coefficient (R) of 0.86 and a percentage mean absolute bias (MABP) of 23.1%. Additionally, the accuracy of TEMPO TOTNO_2_ is found to be highest at midday compared to early-morning or near-sunset hours. The performance also decreases with higher cloud fractions and solar zenith angles.

## 1. Introduction

Tropospheric Emissions: Monitoring of Pollution (TEMPO), launched in April 2023, is the first satellite instrument dedicated to continuous monitoring of air pollutants across North America every daylight hour, delivering exceptional temporal and spatial resolution (Zoogman et al., 2017). Over the past decades, satellite instruments have significantly enhanced our understanding of atmospheric and air pollution dynamics on both regional and global scales by providing crucial data on the quantity, emissions, and transport of pollutants (Edwards et al., 2004; Kaufman et al., 2002; Levelt et al., 2018; Oak et al., 2025; Van Donkelaar et al., 2010). Building on the groundbreaking satellite missions of the early 1970s, this continuous evolution in satellite remote sensing has deepened our understanding of atmospheric chemistry and paved the way for today’s advanced instruments, including TEMPO.

The journey began in the early 1970s with the Backscatter Ultraviolet Radiometer (BUV), which successfully monitored total ozone (O_3_) in the atmosphere (Heath et al., 1973). As an improved version of the BUV instrument, the Solar Backscatter Ultraviolet Radiometer (SBUV) and Total Ozone Mapping Spectrometer were subsequently launched on the Nimbus-7 spacecraft in October 1978 to monitor atmospheric ozone (Heath et al., 1975). Since then, satellite technology has made significant strides. Notable advancements include the launch of the Global Ozone Monitoring Experiment (GOME) 1 in April 1995 aboard ERS-2 satellite (Burrows et al., 1999), followed by the Scanning Imaging Absorption Spectrometer for Atmospheric Cartography (SCIAMACHY) in March 2002 aboard Envisat (Bovensmann et al., 1999), and the Ozone Monitoring Instrument (OMI) in July 2004 aboard NASA’s Aura satellite (Levelt et al., 2018). Subsequent missions further expanded the array of satellite instruments with the GOME-2 aboard the MetOp-A satellite in October 2006 (Munro et al., 2016), the Ozone Mapping Profiler Suite on the Suomi NPP in October 2011, and its subsequent deployments in November 2017 and 2022 on the NOAA-20 and NOAA-21 satellites (Flynn et al., 2014). Other noteworthy additions include GOME-2B and GOME-2C onboard the MetOp-B and MetOp-C satellites in September 2012 and November 2018 (Munro et al., 2016), the Tropospheric Monitoring Instrument (TROPOMI) on Sentinel-5P in October 2017 (Veefkind et al., 2012), the Environmental Trace Gases Monitoring Instrument (EMI) on Gaofen-5 in May 2018 (Zhang et al., 2018), and the Geostationary Environment Monitoring Spectrometer (GEMS) on GEO-KOMPSAT-2B satellite in February 2020 (Kim et al., 2020). While early instruments primarily monitored the ozone layer or a single pollutant like sulfur dioxide (SO_2_), recent advancements now enable the tracking of multiple trace gases including nitrogen dioxide (NO_2_), formaldehyde (HCHO), and SO_2_, thereby vastly expanding the scope of atmospheric studies.

One major pollutant in the Earth’s atmosphere is NO_2_ with significant negative impacts on human health (Chiusolo et al., 2011; Fischer et al., 2015) both directly and through its role in the production of near-surface ozone and particulate matter. As a result, it has been designated a criteria air pollutant monitored and regulated by the Clean Air Act (https://www.epa.gov/clean-air-act-overview). NO_2_ predominantly enters the atmosphere via emissions of nitrogen oxides (NO + NO_2_) through the burning of fossil fuels in vehicles, power plants, and industrial processes (Logan, 1983; Seinfeld & Pandis, 2016). Nitrogen oxides are rapidly oxidized from NO to NO_2_, and NO_2_ is rapidly photolyzed to NO. As NO_2_, nitrogen oxides are rapidly lost via reaction with hydroxyl radical to form nitric acid. The short lifetime of nitrogen oxides of only a few hours results in a highly heterogeneous spatial distribution near anthropogenic emission sources (Judd et al., 2019). This creates complex patterns that are difficult to observe using the limited number of surface stations. For example, only 8% of counties in the United States (251 out of 3,145) reported NO_2_ from a monitor in the Air Quality System of the Environmental Protection Agency (EPA) for 2023.

The high spatial coverage provided by satellite instruments enables the monitoring of air pollution even in areas lacking ground-based stations (Ghahremanloo et al., 2021a). With continuous advancements in satellite technology, the spatial resolution of satellite data has improved from a nominal resolution of 40 × 320 km^2^ from the GOME instrument to 5.5 × 3.5 km^2^ with TROPOMI (Veefkind et al., 2012), and the temporal resolution has been enhanced from every few days to daily. Despite these improvements, the limited number of daily observations from sun-synchronous satellites in the low Earth orbit (LEO) (e.g., TROPOMI) is still inadequate for many applications, such as tracking pollutant transport over a day, fully assessing impacts on human health, or studying the complex chemistry and diurnal dynamics of air quality (Zoogman et al., 2017). Thus, there has been an ongoing demand within the scientific community for more advanced satellite instruments capable of providing a greater frequency of atmospheric observations to support air quality research and applications.

Launched in April 2023, TEMPO provides continuous, hourly daylight measurements of criteria pollutants, including NO_2_, across North America (Zoogman et al., 2017). Operating from geostationary Earth orbit (GEO), TEMPO conducts hourly measurements at a resolution of ∼2 × 4.75 km^2^ to effectively capture the dynamic diurnal cycle of emissions and atmospheric chemistry, a process not observable by the existing LEO satellites that measure only once daily. TEMPO is the North American component of the global GEO constellation for pollution monitoring, working alongside the European Sentinel-4 (launch planned in 2025) and the Korean GEMS with a spatial resolution of 6 × 8 km^2^ (launched 2020) (Kim et al., 2020). This constellation, conducting the first tropospheric trace gas measurements from GEO, extends the legacy of the satellite spectrometers previously deployed in LEO. Hosted on Intelsat-40e (IS-40e), a commercial GEO spacecraft, TEMPO measures spectra that can be used to retrieve a variety of atmospheric components, such as ozone, NO_2_, SO_2_, HCHO, and aerosols, among others (Zoogman et al., 2017).

The enhanced temporal and spatial resolution of TEMPO opens the door to numerous new scientific investigations. For example, TEMPO’s frequent daily data updates are highly advantageous for incorporation into chemical models that aid in air pollution forecasting (Jung et al., 2022) and the refinement of emission inventories (Hsu et al., 2024; Park et al., 2024). TEMPO data is also crucial for exploring the primary emissions from biomass burning and the rapidly evolving chemistry within fire plumes on hourly and daily scales. Additionally, integrating TEMPO data with regional air quality models enables the direct provision of near-real-time pollution reports and forecasts to the public. The broad spatial coverage of TEMPO also supplies invaluable insights for epidemiological studies aimed at understanding the health impacts of air quality. Beyond the United States, TEMPO offers capability to deliver continuous air quality data to more than 99.5% of the Canadian population, a significant benefit given Canada’s vast land area and the limitations of surface monitoring stations (Zoogman et al., 2017). Moreover, TEMPO covers over 78% of Mexico, encompassing the Mexico City area with a population of almost 23 million.

Despite the significant advancements in spatial and temporal resolution that TEMPO offers over prior satellite instruments, a comprehensive investigation to accurately analyze the bias in its NO_2_ product using a diverse array of Pandora instruments is still critically needed. Several studies have validated the data from previous satellite instruments. For instance, Kim et al. (2023) performed an initial validation of GEMS Level-2 total column density of NO_2_ (TOTNO_2_) against Pandora (Herman et al., 2009) ground-based UV-visible spectrometers. Their study, conducted between November 2020 and January 2021 in Seosan, South Korea, revealed a correlation coefficient (R) ranging from 0.62 to 0.78 and a root mean square error (RMSE) between 3.3 × 10^15^ to 5 × 10^15^ molecules/cm^2^, under conditions with cloud fractions below 30%. Similarly, Ghahremanloo et al. (2024) rigorously evaluated GEMS Level-2 (v2) tropospheric column density of NO_2_ (TRPNO_2_) using observations from 22 Pandora stations within the GEMS domain from 2021 to 2023, finding an R of 0.68 and a mean absolute bias (MAB) of 5.7 × 10^15^ molecules/cm^2^. Verhoelst et al. (2021) employed ground-based UV-visible spectrometers to validate TROPOMI NO_2_, including tropospheric, stratospheric, and total column measurements, during the first 2 years of operation. Their findings revealed a bias in TRPNO_2_ of 23%–37% under clean to slightly polluted conditions, and a modest median bias of 0.2 × 10^15^ molecules/cm^2^ in the stratospheric NO_2_ measurements. For the TOTNO_2_, the bias ranged from zero to +50%. Additionally, Judd et al. (2020) assessed the accuracy of TROPOMI TRPNO_2_ measurements using airborne platforms and surface Pandora stations in 2018 in New York, noting that TROPOMI TRPNO_2_ measurements showed a higher correlation with airborne data (R: 0.98) compared to data from Pandora stations (R: 0.92).

This study provides a comprehensive analysis of the bias in TEMPO TOTNO_2_ data by leveraging Pandora observations from the Pandonia Global Network (PGN). PGN provides standardized, calibrated, and quality assured TOTNO_2_ data over the globe. The study covers the period from TEMPO’s initial data release on 2 August 2023, to 31 December 2024. We aim to thoroughly evaluate TEMPO TOTNO_2_ and explore how various factors, such as cloud cover, solar zenith angle (SZA), differing NO_2_ levels, observation times, and the geographical positioning of Pandora stations, influence its measurement accuracy. We also use Pandora observations to validate TOTNO_2_ measurements from TEMPO, TROPOMI, and OMI at the overpass times of TROPOMI and OMI. This comparative validation was performed using both the original resolution of each instrument and a version regridded to OMI’s resolution, in order to assess the impact of spatial resolution on the accuracy of TOTNO_2_ measurements. This direct comparison conducted at identical locations and times, will enhance users’ understanding of the measurement accuracy across all three instruments.

## 2. Study Area and Data

### 2.1. TEMPO

**Figure 1.**
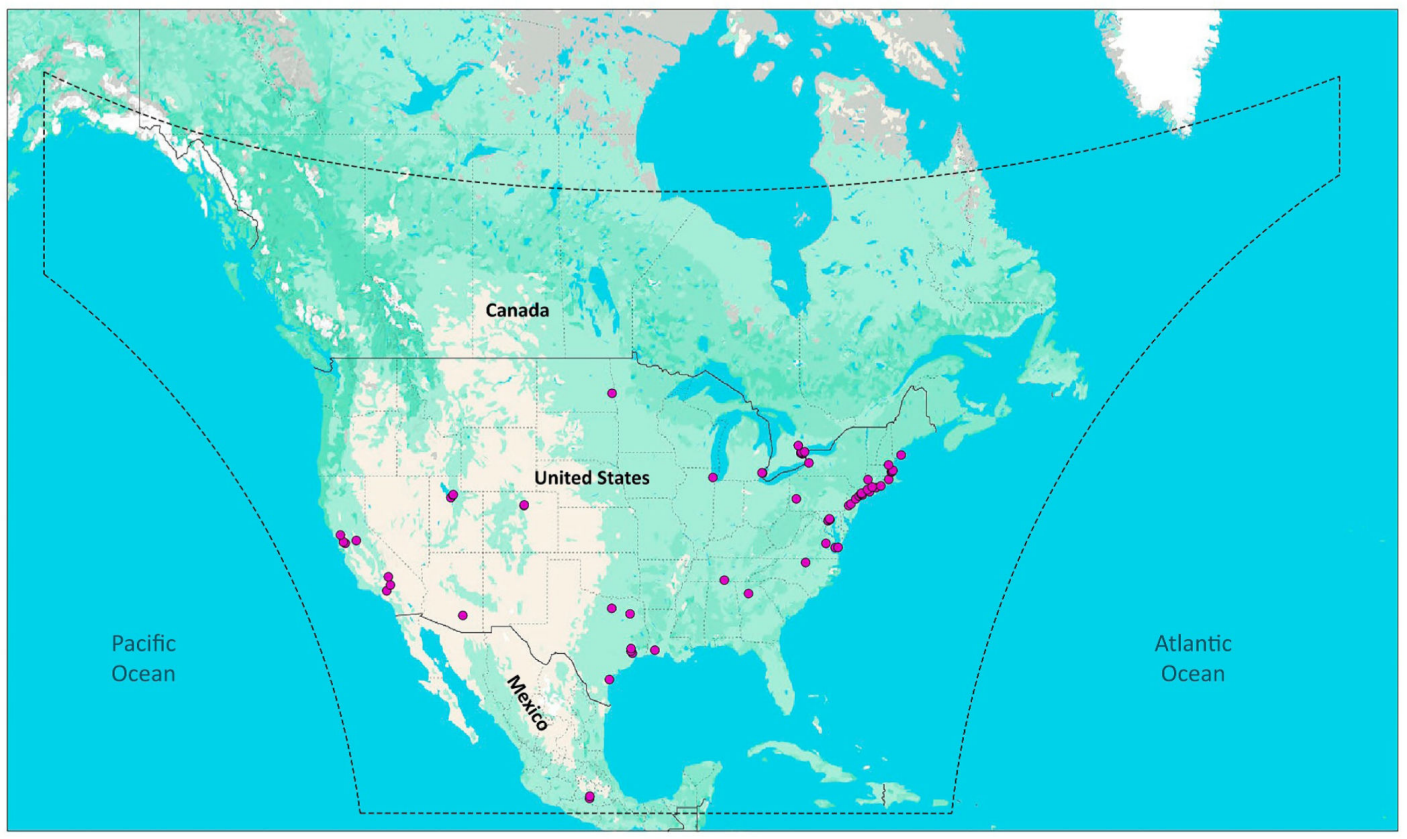
North America and the TEMPO field of regard (FOR) (study area): The dashed line delineates the TEMPO FOR. Purple circles indicate the locations of Pandora stations from the Pandonia Global Network (PGN) observing total column density of NO_2_ (TOTNO_2_). The study period is from 02 August 2023 (TEMPO’s first light) to 31 December 2024.

TEMPO, a GEO instrument installed on the IS-40e commercial satellite, was launched on 7 April 2023, to observe Earth’s atmosphere across North America. It boasts a high spatial resolution of 2 × 4.75 km^2^ at center of field of regard (FOR) and captures hyperspectral images at least every daylight hour in 1,181 East/West mirror steps with 2,036 North/South ground pixels. TEMPO achieved its Earth-view first light on 2 August 2023, and started normal operations following a successful post-launch acceptance review in October 2023 (González Abad et al., 2024). The instrument collects spectral data critical for analyzing key atmospheric constituents such as ozone, NO_2_, SO_2_, HCHO, glyoxal (C_2_H_2_O_2_), bromine monoxide (BrO), iodine monoxide (IO), water vapor, aerosols, cloud parameters, ultraviolet (UV) radiation, and vegetative properties. Scans by TEMPO, moving from East to West, are usually completed within an hour but may last about 40 min during early morning and late evening with fewer East/West mirror steps. During infrequent special observations, even higher temporal resolution (<10 min) is occasionally available when the covered East-West region is reduced. TEMPO’s nominal FOR, as depicted by the dashed line in Figure 1, ranges from approximately 18°N to 63°N in latitude and 150°W to 30°W in longitude, and covers the majority of the North American continent, including the entire contiguous United States (CONUS), roughly 78% of Mexico, and a substantial portion of Canada—encompassing about 99% of its population. It also includes Cuba, the Dominican Republic, Haiti, and Jamaica, as well as Puerto Rico and several other smaller islands and regions.

The TEMPO instrument features a UV-visible spectrometer equipped with two 2D 2,048 × 1,028 charge-coupled device detectors situated on a single focal plane. These detectors capture two distinct spectral bands: one spanning approximately 293–494 nm and the other roughly 538–741 nm. The 2,048 dimension is aligned with the spatial axis, while the 1,028 dimension corresponds to the spectral axis. The TEMPO spectrometer’s slit is oriented along the North/South (N/S) axis, allowing for the simultaneous measurement of 2,048 spatial pixels across the N/S or across-track direction. Each spectral band comprises 1,028 spectral pixels. The instrument achieves a spectral resolution of about 0.6 nm at full width at half maximum (FWHM) with a spectral interval of approximately 0.2 nm (Nowlan et al., 2025). It is important to note that while TEMPO captures NO_2_ data across both the UV and visible spectral bands, GEMS, another GEO satellite instrument, specifically targets only the shorter wavelengths from 300 to 500 nm, for its NO_2_ observations. Nominal TEMPO hourly observations are divided into nine granules, each capturing approximately 6.7 min of data. Owing to the constant Instantaneous Field of View, the ground pixel size varies based on the viewing zenith angle. At the center of the FOR, the ground pixel size measures about 2 × 4.75 km^2^. However, this footprint reduces to ∼8 km^2^ over Mexico City and expands to ∼21 km^2^ over the Canadian oil sands (Nowlan et al., 2025). Further information on the instrument is available in Zoogman et al. (2017) and the TEMPO NO_2_ Algorithm Theoretical Basis Document (Nowlan et al., 2025).

The TEMPO retrieval algorithm used to produce TOTNO_2_ has three major processing steps (Nowlan et al., 2025). TEMPO TOTNO_2_ is derived by first calculating the slant column density (SCD) of NO_2_ using backscattered solar light across part of the visible spectral range, specifically using wavelengths of 405–465 nm. This is followed by calculating the air mass factors (AMFs) needed to compute the vertical column densities (VCDs). The GEOS-CF chemical forecast (Keller et al., 2021) with the spatial resolution of 0.25° × 0.25° is used as a priori NO_2_ profiles for the TEMPO AMF calculation. These AMFs account for the path length of photons traveling through the atmosphere, which varies with geometry and atmospheric conditions. The final step involves applying the AMFs and separating the stratospheric and tropospheric components of NO_2_ VCDs using additional ancillary data and modeling inputs as described in Nowlan et al. (2025) using the method of Geddes et al. (2018).

The sources of uncertainty in the TEMPO TOTNO_2_ measurements stem from several identified issues, as highlighted by González Abad et al. (2024). Cloud coverage notably affects the accuracy and precision of TOTNO_2_ retrievals; inhomogeneities caused by partially cloudy pixels can significantly reduce NO_2_ retrieval performance. Additionally, the cloud retrieval algorithm often overestimates cloud fractions in clear or nearly clear sky pixels due to a known overestimation in the TEMPO version 3 radiances (Wang et al., 2025). When cloud pressure data is unavailable from the cloud retrieval, a climatological value is substituted, which can lead to significant biases in AMF calculations. Another potential source of bias is the presence of aerosols in the atmosphere. Large SZA also decrease retrieval accuracy, adversely affecting TOTNO_2_ measurements. Moreover, the accuracy of Geometry-dependent Lambertian Equivalent Reflectivity (GLER) values, derived from the Moderate Resolution Imaging Spectroradiometer (MODIS), might be compromised in some TEMPO scans that are distant from MODIS overpass times. This temporal misalignment can influence AMF accuracy and introduce spatial inconsistencies and biases in NO_2_ and cloud fraction data.

In this study, we utilized TEMPO Level-3 v3 data (TEMPO_NO2_L3_V03: NASA/LARC/SD/ASDC, 2024), which is generated from all granules in a single scan using an area-weighted gridding technique to determine NO_2_ on a 0.02° × 0.02° regular grid. This algorithm maps the corresponding Level-2 data onto a uniform 0.02° × 0.02° grid. Importantly, the gridding process is conducted on all available data without filtering for quality flags, cloud cover, etc. Because Level-3 grid has a finer spatial resolution than the original TEMPO ground pixels, the data precision provided in the Level-3 files is very close to that from the Level-2 files, and the accuracy is also expected to be similar. As suggested in the TEMPO user guide, the same usage and filtering recommendations applicable to Level-2 data are also applied to the Level-3 data set (González Abad et al., 2024).

For most of the analyses, we exclusively incorporated data that carried a “main_data_quality_flag” of zero, ensuring the exclusion of suspect measurements and low-quality pixels. Additional filters were applied to assess the impact of each filtering scenario on the accuracy of TEMPO TOTNO_2_ and will be discussed in Section 4. A total of 7,135 TEMPO scans from 2 August 2023, to 31 December 2024 were obtained from the NASA Langley Atmospheric Science Data Center (https://asdc.larc.nasa.gov/). It is important to note that we did not use TOTNO_2_ data in the “support_data” group of TEMPO Level-3 files in this study. To derive the TEMPO TOTNO_2_ data, we added the TRPNO_2_ and stratospheric NO_2_ column densities from the “product” group of TEMPO Level3 files. This is due to the fact that the TOTNO_2_ data in the “support_data” group of TEMPO product files is calculated using the full NO_2_ model profile from the surface to the top of the atmosphere, therefore, it retains more influence of the a priori profile as the troposphere and stratosphere are not able to be determined independently, and its use is discouraged (González Abad et al., 2024; Nowlan et al., 2025).

### 2.2 Pandora

PGN is an international collaborative effort established to provide vital measurements for the validation of satellite data and the advancement of atmospheric studies. As a collaborative effort between NASA and the European Space Agency (ESA), PGN employs ground-based Pandora spectrometers to monitor various trace gases, such as NO_2_, ozone, SO_2_, and HCHO. It is also worth mentioning that Environment and Climate Change Canada and the United States EPA have partnered with NASA and ESA in the deployment of Pandora instruments across North America, under the TEMPO FOR (Szykman et al., 2019). The data produced by PGN are systematically standardized, calibrated, and made accessible to the public, thereby aiding scientific research and applications in atmospheric science.

We obtained Pandora TOTNO_2_ data from 65 Pandora stations from the PGN (https://www.pandonia-globalnetwork.org/) from 2 August 2023, to 31 December 2024. Pandora observations of TOTNO_2_ were used to quantify the bias in TOTNO_2_ measurements from TEMPO, TROPOMI, and OMI in this study. Figure 1 shows the locations of the 65 Pandora stations, which are indicated in purple. However, it excludes stations that did not record high-quality observations during the study period. We also excluded the Pandora station installed at the CN Tower in Toronto, Canada, from the analyses presented in Sections 4.1–4.4 and Section 4.6. This exclusion was due to the station’s high-altitude installation—approximately 330 m above ground level—and the detailed reasoning behind this decision is provided in Section 4.5. However, we retained this station in the analysis conducted in Section 4.5, which specifically examines the relationship between TEMPO and Pandora measurements at individual stations. Table S1 in Supporting Information S1 provides details on Pandora stations used in this study and the time periods during which each station measured TOTNO_2_ levels.

Pandora spectrometers utilize two primary modes for measurements: Direct-Sun and Multi-Axis Differential Optical Absorption Spectroscopy (MAX-DOAS) (Alwarda et al., 2025). In the Direct-Sun mode, the instruments measure solar irradiance directly, enabling the accurate quantification of total column densities of atmospheric trace gases, such as NO_2_, through the analysis of absorption signatures in the sunlight traversing the Earth’s atmosphere. This method is particularly precise because of high signal due to the precise tracking of the sun’s path and the minimal uncertainties related to the AMF from scattered light. On the other hand, the MAX-DOAS mode captures scattered sunlight at multiple elevation angles, increasing the sensitivity to varying concentrations of trace gases at different altitudes and facilitating the derivation of vertical atmospheric profiles (Alwarda et al., 2025; Frieß et al., 2019; Verhoelst et al., 2021).

However, the MAX-DOAS mode introduces significant uncertainties due to factors like aerosol interactions and the complexities involved in radiative transfer modeling needed for interpreting the data. The precision of Pandora’s Direct-Sun TOTNO_2_ measurements under clear-sky conditions is 0.01 Dobson Units (DU) for the slant column, equating to about 2.69 × 10^14^ molecules/cm^2^, with a nominal accuracy of 0.1 DU for the vertical column at a 2σ confidence level, or roughly 2.69 × 10^15^ molecules/cm^2^ (Herman et al., 2009). The PGN documentation (Cede et al., 2025) also provides an uncertainty quantification as a function of SZA (10°–85°) (Figure 9 in Cede et al., 2025). The uncertainty peaks at SZA of 10°, with a median value of 0.5 × 10^15^ molecules/cm^2^ and an upper bound of 1.5 × 10^15^ molecules/cm^2^. According to their analyses, the uncertainty decreases as the SZA increases. In this research, the TOTNO_2_ data employed were obtained using the Direct-Sun mode. We specifically chose Pandora TOTNO_2_ observations that were marked with quality flags of 0 or 10, ensuring only high-quality data were included. Observations deemed to be of low or medium quality were systematically excluded from our analysis.

### 2.3. TROPOMI

In this research, we compiled a total of 5,162 TROPOMI TOTNO_2_ overpasses (reprocessed/offline [RPRO/OFFL] Level-2 v2 product) (Copernicus Sentinel data, 2021; Van Geffen et al., 2022) with a nadir resolution of 5.5 × 3.5 km^2^, covering the period from 2 August 2023 to 31 December 2024 in the TEMPO domain. These orbits were sourced from the NASA Goddard Earth Sciences Data and Information Services Center (GES-DISC), available at (https://disc.gsfc.nasa.gov/). Launched in October 2017 aboard the Sentinel-5 Precursor satellite, TROPOMI (Veefkind et al., 2012) orbits the Earth in a sun-synchronous path at an altitude of 824 km, crossing the equator daily at 13:30 local time. This advanced instrument plays a key role in tracking atmospheric pollutants including ozone, methane, carbon monoxide, HCHO, NO_2_, SO_2_, and aerosols. To maintain high data quality, we implemented a filtering protocol that discarded any pixels with a quality assurance (QA) threshold below 0.75, and excluding observations compromised by snow, ice, SZA higher than 70°, or problematic retrievals. Pixels with cloud fraction greater than 15% were also excluded to ensure the reliability of our analyses. Similar to TEMPO TOTNO_2_, we added the TRPNO_2_ and stratospheric NO_2_ column densities to obtain TROPOMI TOTNO_2_.

### 2.4. OMI

OMI was deployed on NASA’s Aura satellite in July 2004 and is a nadir-viewing spectrometer that measures solar backscattered radiation within the UV and visible spectra (Levelt et al., 2018). It offers daily global monitoring with a spatial resolution of 13 × 24 km^2^ at nadir, facilitating detailed monitoring of atmospheric trace gases including ozone, NO_2_, SO_2_, and HCHO, in addition to aerosols and cloud characteristics. Since 2007, OMI has been affected by a “row anomaly,” attributed to an internal blockage impacting around half of its 60 viewing angles (Torres et al., 2018). This issue has resulted in diminished spatial coverage and data gaps, adversely affecting the accuracy of various retrieved atmospheric parameters, such as NO_2_. Variations in the row anomaly post-2007, along with striping and instrumental radiometric degradation, have introduced an annual increase in uncertainty of 1%–2% for the OMI NO_2_ SCD, and a reduction in coverage area (Schenkeveld et al., 2017; Zara et al., 2018). A total of 5,159 OMI TOTNO_2_ Level-2 v4 overpasses (Krotkov et al., 2019; Lamsal et al., 2020) were acquired from the GES-DISC (https://disc.gsfc.nasa.gov/) in this study. To enhance data quality, pixels compromised by low quality flags, the row anomaly, cloud coverage exceeding 15%, and SZA higher than 70° were excluded. Similar to TOTNO_2_ in TEMPO and TROPOMI, we added the TRPNO_2_ and stratospheric NO_2_ column densities to obtain OMI TOTNO_2_.

## 3. Methodology

To quantify and analyze the bias in TEMPO TOTNO_2_ data using Pandora TOTNO_2_ observations from 2 August 2023 to 31 December 2024, we calculated averages of Pandora TOTNO_2_ observations recorded within a 15-min window before and after the TEMPO measurements, producing 118,221 matched TEMPO-Pandora data pairs. These pairs encompass data across all cloud fraction levels, though their number may decrease depending on the evaluation scenarios and filtering thresholds applied in Section 4. Spatially, TEMPO pixels were aligned with Pandora stations within those same pixels. This research also undertook a comparative validation of TOTNO_2_ measurements from TEMPO, TROPOMI, and OMI instruments against Pandora data to evaluate the accuracy of each satellite instrument in measuring TOTNO_2_. This involved extracting all TEMPO TOTNO_2_ measurements taken within 15 min before or after the overpass times of the TROPOMI and OMI instruments, ensuring spatial and temporal alignment with Pandora observations. Notably, TROPOMI and OMI instruments pass over the equator daily at approximately 13:30 local time.

We employed several evaluation metrics to analyze the bias in the TOTNO_2_ data from TEMPO, TROPOMI, and OMI instruments using Pandora observations. These metrics include the R, index of agreement (IOA), MAB, mean bias (MB: satellite data minus Pandora observations), and RMSE. Additionally, we calculated the percentage MB (MBP), percentage MAB (MABP), and percentage RMSE (RMSEP) by normalizing each metric against the mean TOTNO_2_ value from Pandora. The mathematical formulas for these metrics are defined in Equations 1–5. R measures how closely two sets of data—satellite TOTNO_2_ and Pandora observations—move together, indicating their linear relationship without considering whether they are biased or off by a consistent amount. In contrast, IOA not only assesses how closely the values follow each other but also checks how similar their actual sizes or amounts are (Legates and McCabe, 1999; Willmott, 1981). Lower values of R and IOA indicate lesser agreement between TEMPO TOTNO_2_ and Pandora observations. Additionally, MB, MAB, and RMSE quantify the bias in TEMPO TOTNO_2_ data relative to Pandora data. A key distinction is that RMSE weights larger errors more heavily by squaring the error values, thus always exceeding MAB values. MAB reflects the total absolute bias without considering the direction of errors, whereas MB allows for the cancellation of positive and negative errors, specifying degree of overestimations or underestimations in TOTNO_2_ measurements (Ghahremanloo et al., 2021b, 2021c, 2023).

(1) $$R=\frac{\sum_{i=1}^{n}\left(E_{i}-\bar{E}\right)\cdot \left(O_{i}-\bar{O}\right)}{\sqrt{\sum_{i=1}^{n}{\left(E_{i}-\bar{E}\right)}^{2}}\cdot \sqrt{\sum_{i=1}^{n}{\left(O_{i}-\bar{O}\right)}^{2}}}$$

(2) $$\text{IOA}=1-\frac{\sum_{i=1}^{n}{\left(E_{i}-O_{i}\right)}^{2}}{\sum_{i=1}^{n}{\left(\left|E_{i}-\bar{O}\right|+\left|O_{i}-\bar{O}\right|\right)}^{2}}$$

(3) $$\text{MAB}=\frac{1}{N}\sum_{i=1}^{n}\left|E_{i}-O_{i}\right|$$

(4) $$\text{MB}=\frac{1}{N}\sum_{i=1}^{n}\left(E_{i}-O_{i}\right)$$

(5) $$\text{RMSE}=\sqrt{\frac{1}{N}\sum_{i=1}^{n}{\left(E_{i}-O_{i}\right)}^{2}}$$

where $E_{i}$ is TOTNO_2_ data measured using TEMPO, TROPOMI, or OMI instrument; $O_{i}$ the observed TOTNO_2_ data from Pandora; $\bar{E}$ the mean TOTNO_2_ data from the satellite instrument; $\bar{O}$ the mean Pandora TOTNO_2_; and N is number of samples.

In this study, we conducted a comprehensive analysis to quantify the bias in TEMPO TOTNO_2_ data, examining the impact of various factors on its performance. Initially, we explored the influence of different cloud fraction filtering strategies on TEMPO TOTNO_2_ performance and also assessed its accuracy across pixels with varying cloud cover ranges. This helps to give users a clearer perspective on the impact of cloud fraction filtering on TEMPO TOTNO_2_ and they gain insights into selecting thresholds that best suit their data quality needs—balancing reliable measurements with broader spatial coverage. Subsequently, we made a thorough examination to see how well TEMPO measures TOTNO_2_ levels across different ranges of TOTNO_2_. This ensures the sensor’s retrieval performance across various atmospheric NO_2_ regimes, from relatively clean environments to heavily polluted regions. We also evaluated the accuracy of TEMPO TOTNO_2_ across different SZA ranges. The quality of high SZA measurements is affected by lower signal which increases spectral fitting uncertainties, as well as significant errors introduced in the AMF by surface reflectance assumptions and cloud measurements at extreme angles, and the reduced sensitivity of scattering weights near the surface (Nowlan et al., 2025). An assessment of TEMPO accuracy at a range of SZAs helps to better understand how geometry-driven uncertainties may affect the TOTNO_2_ measurements, helping users refine analyses under specific SZA thresholds.

Moreover, we investigated the effect of local observation hour on TEMPO TOTNO_2_ accuracy. Assessing TEMPO bias at different times of day provides a clearer understanding of how time-dependent processes, such as planetary boundary layer (PBL), influence measurement reliability. We then quantified the bias in TEMPO TOTNO_2_ at each of 65 Pandora stations and analyzed reasons for discrepancies in TEMPO performance among different sites. Examining performance at each station reveals location-specific discrepancies and helps address regional factors that could affect retrieval accuracy. Finally, we obtained TEMPO TOTNO_2_ data measured during the overpass times of TROPOMI and OMI, conducting a comparative validation of all three instruments against Pandora observations to assess each sensor’s ability to measure TOTNO_2_ accurately. This comparative validation was performed using both the original resolution of each instrument and a version regridded to OMI’s resolution, in order to assess the impact of spatial resolution on the accuracy of TOTNO_2_ measurements. This intercomparison highlights each instrument’s ability to measure TOTNO_2_ under shared atmospheric conditions, offering a broader context for future applications. Together, these scenarios provide a more complete picture of TEMPO’s measurement capabilities and guide both researchers and practitioners in optimally leveraging the data for air quality monitoring and research.

## 4. Results and Discussion

### 4.1. Impact of Cloud Cover on TEMPO TOTNO_2_

The TEMPO cloud product (CLDO4) (Wang et al., 2025) provides cloud pressure and cloud fraction, both essential for calculating AMF in TEMPO trace gas retrievals and for removing cloud-contaminated measurements. AMFs are used to convert the SCDs obtained from spectral fitting to VCDs using the formula VCD = SCD/AMF. By definition, an AMF describes the optical path sunlight follows from the Sun to the Earth’s surface or clouds and back to the satellite sensor. It depends on factors such as surface reflectance, vertical distributions of atmospheric absorbers and scatterers, and the geometric configuration of illumination and observation (Palmer et al., 2001). The TEMPO NO_2_ product provides two types of cloud fraction: Cloud radiance fraction and effective cloud fraction. In this study, we examined the performance as a function of the effective cloud fraction, which has been described by Wang et al. (2025) and used for the recommended cloud filtering thresholds in the TEMPO user guide (Gonzalez Abad et al., 2024).

Cloud coverage significantly impacts the accuracy and precision of TOTNO_2_ retrievals. The version-3 TEMPO v3 NO_2_ retrievals show an overestimation of 0.05 in the effective cloud fraction calculated in the cloud algorithm, particularly in clear or nearly clear sky pixels, as identified by Wang et al. (2025). This overestimation is likely to cause inaccuracies in the final trace gas outputs under clear sky conditions. Such errors are primarily due to the effects of a high bias in the version-3 L1B calibrated radiances, as well as inaccuracies in the GLER, both influencing cloud fraction assessments. Additionally, inaccuracies in GLER impact the trace gas measurements by causing errors in the computation of scattering weights (Nowlan et al., 2025). Generally, this high bias in cloud fraction is expected to lead to an overestimation of the NO_2_ column, especially in areas with high pollution levels. It is also important to highlight that the performance of the version-3 TEMPO NO_2_ spectral fitting decreases over partially cloudy pixels because of the inhomogeneous illumination of the instrument slit. This results in significant fitting uncertainties in the retrieved SCDs for these partially cloudy scenarios (Nowlan et al., 2025). Therefore, it is crucial to analyze the impact of cloud cover on TEMPO TOTNO_2_.

**Figure 2.**
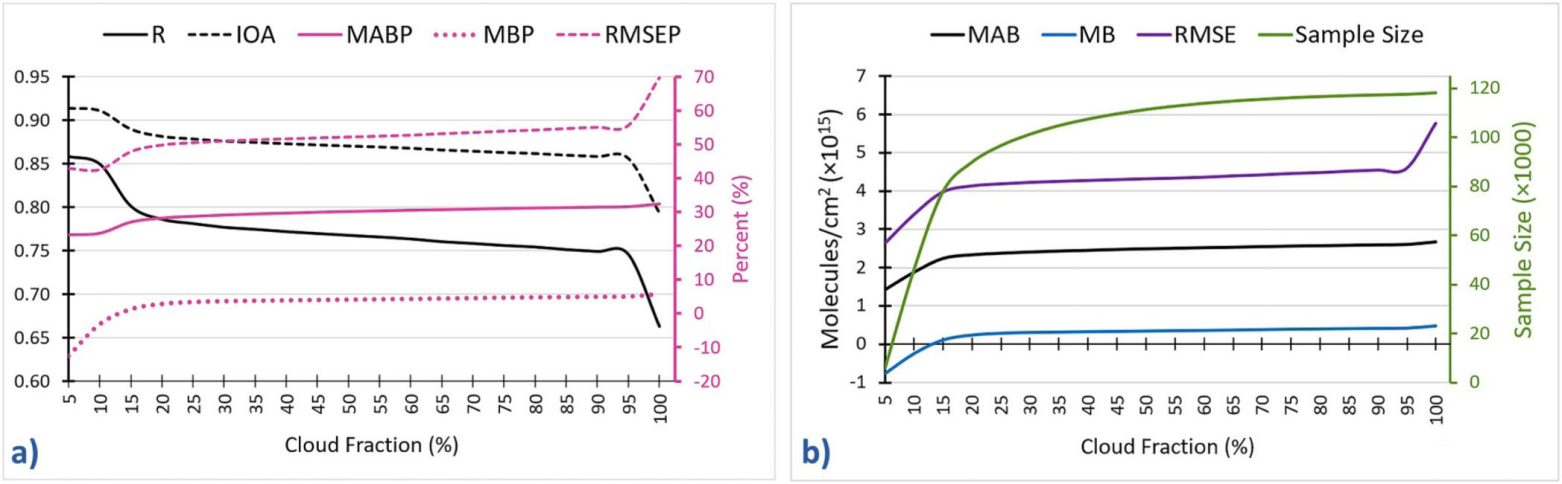
The accuracy of TEMPO TOTNO_2_ under varying cloud fraction filtering thresholds, compared against Pandora observations. The validation metrics are the correlation coefficient (R), the index of agreement (IOA), the mean absolute bias (MAB), the mean bias (MB: TEMPO minus Pandora), the root mean square error (RMSE), and the percentage biases (MABP, MBP, and RMSEP), which are MAB, MB, or RMSE divided by mean Pandora TOTNO_2_.

Figure 2 illustrates the performance of TEMPO TOTNO_2_ under varying cloud fraction filtering thresholds, compared against Pandora observations. For these analyses, we excluded TOTNO_2_ measurements where the SZA exceeded 70°, and the “main_data_quality_flag” were not equal to zero. In this scenario, cloud fraction levels tested ranged from 0% to 100%. To calculate the evaluation metrics for each threshold, pixels with cloud fractions exceeding the threshold were excluded. For example, in the case of a 5% cloud fraction filter, only pixels with a cloud fraction of 5% or lower were considered for validation. According to Figure 2, the best performance of TEMPO TOTNO_2_ was observed at a cloud fraction threshold of 5%, where the R and IOA reached 0.86 and 0.91, respectively. Additionally, MAB, MB, and RMSE were calculated as 1.4 × 10^15^, − 7.6 × 10^14^, and 2.6 × 10^15^ molecules/cm^2^, respectively. At this threshold, TEMPO TOTNO_2_ also achieved an MABP of 23.1% and an RMSEP of 42.9%, with an underestimation of 12% against Pandora observations, indicating high accuracy in measuring TOTNO_2_. Beyond this threshold, both the R and IOA values began to decline, while all bias metrics, with the exception of MB, showed an increase. A notable decline in TEMPO TOTNO_2_ performance was observed in the 95%–100% cloud fraction range. Interestingly, TEMPO tends to underestimate TOTNO_2_ at thresholds lower than ∼13% and overestimate it at thresholds higher than ∼13%. As expected, the number of TEMPO– Pandora pairs increased with higher cloud fraction thresholds (Figure 2b). Specifically, the data set contained 5,335 pairs at 5% and 45,664 pairs at 10%, eventually reaching 118,221 pairs at 100%.

Figure S1 in Supporting Information S1 also displays the performance of TEMPO TOTNO_2_ across distinct cloud cover ranges, without the application of any cloud fraction filtering. The histogram in Figure S1a in Supporting Information S1 indicates that the majority of TEMPO-Pandora TOTNO_2_ pairs occur within a cloud fraction range of 5%–20%. The sample size in other cloud fraction ranges is considerably smaller, partly because some TEMPO-Pandora pairs with higher cloud fractions have been excluded during the filtering of low-quality Pandora observations, even though we did not directly apply cloud filtering to prepare Figure S1 in Supporting Information S1. Additionally, the geographical location and associated climate conditions of the Pandora stations represent another crucial factor. As expected, the bias in TEMPO TOTNO_2_ data increases with higher cloud fractions (Figure S1 in Supporting Information S1), while R and IOA generally decrease. According to Figure S1 in Supporting Information S1, there is a marginal difference in the accuracy of TEMPO TOTNO_2_ between cloud fraction ranges of 0%–5% and 5%–10%. TEMPO data in cloud fraction levels of 5%–10% have the highest accuracy (R: 0.85; IOA: 0.91; MAB: 1.940 × 10^15^ molecules/cm^2^; MABP: 23.7%) while the TOTNO_2_ data measured in the cloud fraction range of 95%–100% have the lowest accuracy (R: 0.62; IOA: 0.43; MAB: 9.795 × 10^15^ molecules/cm^2^; MABP: 128%). Although TEMPO tends to underestimate TOTNO_2_ in lower cloud fractions (0%–10%), it overestimates it in higher fractions. Specifically, TEMPO has an underestimation of 13% in the cloud fraction range of 0%–5% but overestimates TOTNO_2_ by 80% in the 95%–100% range.

The impact of higher cloud fractions on the accuracy of TEMPO TOTNO_2_ observed in this study aligns with findings from previous research. Kim et al. (2023) assessed the performance of GEMS TOTNO_2_ relative to Pandora TOTNO_2_ at various cloud fraction levels across four Pandora stations in South Korea. They reported significant difference in the accuracy of GEMS TOTNO_2_ at various cloud fraction filtering thresholds in four stations: 30% threshold (R: 0.62 to 0.78; RMSE: 3.3 × 10^15^ to 5 × 10^15^ molecules/cm^2^), 50% threshold (R: 0.42 to 0.53; RMSE: 3.8 × 10^15^ to 5.5 × 10^15^ molecules/cm^2^), 70% threshold (R: 0.35 to 0.48; RMSE: 4.7 × 10^15^ to 5.5 × 10^15^ molecules/cm^2^). Geddes et al. (2012) also reported significant decrease in the agreement of OMI TRPNO_2_ with ground-level NO_2_ as cloud fraction increased to 100%. According to their study, when cloud fractions are below approximately 60%, OMI TRPNO_2_ mainly rely on the measurement itself, surface reflectivity, and the shape of the a priori profile. As the cloud fraction approaches 100%, the TRPNO_2_ measurements increasingly depend on the a priori profile below the cloud tops and are less dependent on the measurement.

Figures 2 and S1 in Supporting Information S1 illustrate that, even at very high cloud fraction ranges (e.g., 95%–100%), a persistent correlation (R: 0.62) exists between TOTNO_2_ values measured by TEMPO and Pandora (see Figure S1b in Supporting Information S1). This phenomenon may be attributed to the varying opacity of clouds; not all clouds completely block sunlight (Nouri et al., 2019). Additionally, as cloud fraction approaches 100%, the TEMPO TOTNO_2_ measurements increasingly rely on the a priori NO_2_ profile rather than direct NO_2_ measurements (Geddes et al., 2012). Since the a priori NO_2_ profile is derived from modeled estimates of NO_2_ levels, the retrieved TOTNO_2_ will continue to show some degree of agreement with Pandora observations. Lastly, as clouds are typically found in the troposphere, up to about 12 km above the Earth’s surface (Huang et al., 2012), stratospheric NO_2_ remains unobstructed by clouds. Thus, TEMPO can detect stratospheric NO_2_ irrespective of cloud cover, contributing to the observed correlation (R: 0.62) with Pandora’s measurements that encompass both tropospheric and stratospheric NO_2_.

It is important to clarify how Pandora can provide high-quality TOTNO_2_ observations even when TEMPO indicates a cloudy scene. Pandora retrievals are subject to strict internal noise thresholds, and data are accepted only when the spectral fit noise is below these limits, or excluded if the measurements are impacted by “unwanted” spectral signals (Cede, 2024). In this study, we included only Pandora observations with quality flags of 0 or 10 to ensure the use of high-quality data. This should remove instances when the instrument does not retrieve a high-quality measurement due to a larger than acceptable diffuse light fraction, as would be the case under thick cloud cover. Herman et al. (2009) demonstrated that Pandora achieves a precision of approximately 0.01 DU under clear-sky conditions. Even in moderately cloudy conditions or thin clouds, a drop in signal has minimal impact on the slant column fit thanks to Pandora’s high signal-to-noise ratio (SNR) and rapid millisecond-level integration times. Under thicker cloud cover, the SNR decreases as fewer photons reach the sensor, degrading precision to around ±0.2 DU. Moreover, the Pandora’s Direct-Sun “pencil beam” has a narrow 1.6° FOV (Herman et al., 2009), allowing it to sample only the solar disc. In contrast, a single TEMPO pixel spans several square kilometers. This spatial mismatch means Pandora can detect sunlight through sub-pixel cloud gaps or transient clearings that are not resolved in TEMPO observations. As a result, even when TEMPO reports an overcast pixel, Pandora may still receive sufficient direct sunlight to retrieve high-quality NO_2_ measurements.

It is worth noting that satellite retrievals of NO_2_ column densities below clouds often use the Independent Pixel Approximation to estimate NO_2_ that lies under clouds in each pixel. In this approach, each pixel is treated as a combination of a clear-sky portion and a fully opaque cloudy part. The NO_2_ column beneath the clouds— unobservable to the satellite—is reconstructed using a model-derived ghost column and added back before applying mixed AMFs (Boersma et al., 2011). This framework, adopted operationally by several satellite instruments, enables near-global coverage but introduces uncertainty due to reliance on modeled NO_2_ vertical profiles. In contrast, Martin et al. (2002) proposed a different retrieval strategy that is also used by TEMPO. They divided each satellite pixel into a cloudy slice and a clear-sky portion, measured the cloud optical thickness, and used a radiative transfer model to simulate the amount of light transmitted through each slice. By combining the two modeled contributions, they retrieved the NO_2_ column without relying on a model-based ghost column to account for the NO_2_ hidden beneath the clouds. Retrieving NO_2_ data below clouds offers several advantages. Most notably, it reduces data gaps and missing values in satellite data. By analyzing surface monitors, Goldberg et al. (2025) showed that afternoon NO_2_ is on average 36% higher on cloudy days than on clear ones, and even the daily mean is 17% higher when you include cloudy days instead of restricting to clear skies. Ignoring under-cloud columns therefore leads to a consistent low bias in annual-mean NO_2_ maps.

### 4.2 Performance of TEMPO TOTNO_2_ for Different TOTNO_2_ Levels

**Figure 3.**
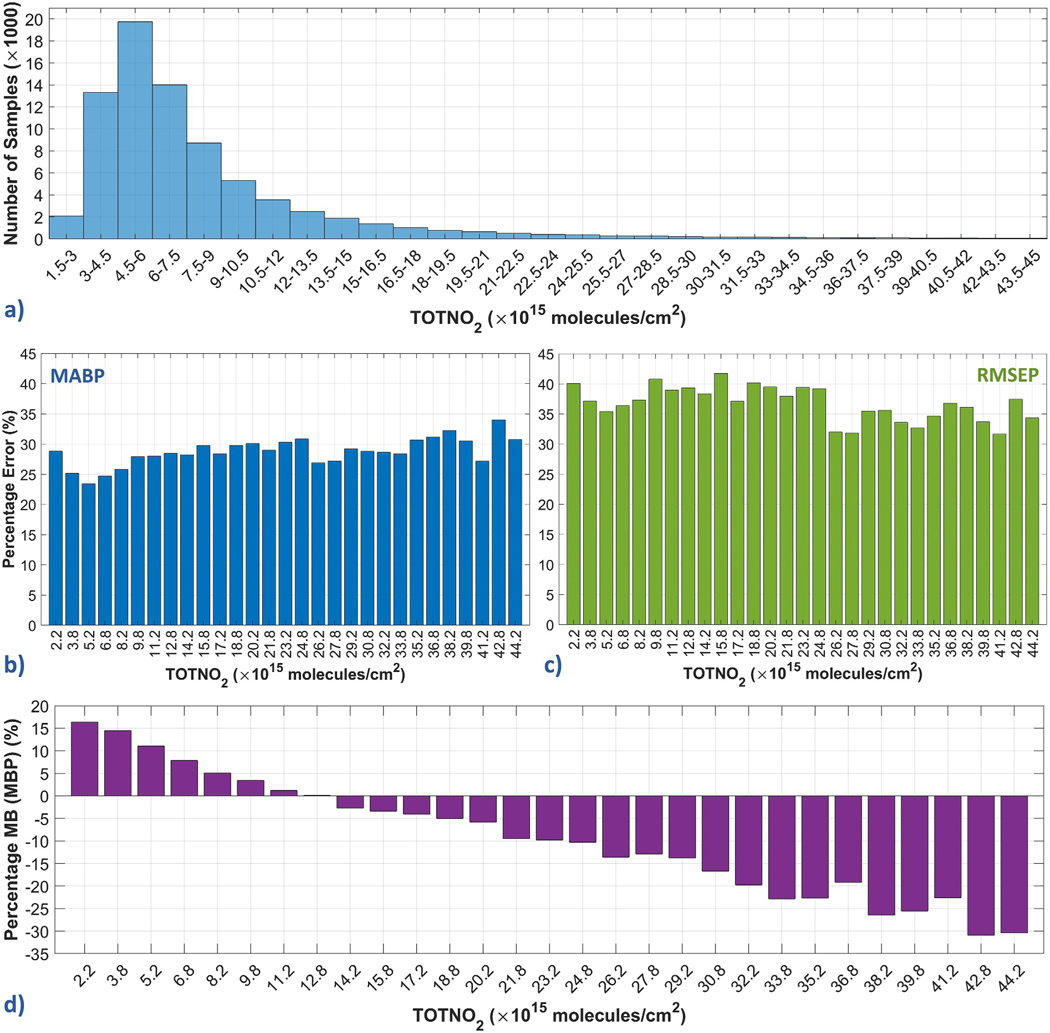
Performance of TEMPO TOTNO_2_ across different NO_2_ levels: (a) histogram of TEMPO TOTNO_2_, (b) percentage MAB (MABP), (c) percentage RMSE (RMSEP), and (d) percentage MB (MBP). The histogram excludes bins with TOTNO_2_ values greater than 45 × 10^15^ molecules/cm^2^ where sample sizes are fewer than 40. All subplots in this figure use the same TOTNO_2_ bin ranges. In panels (b–d), the *X*-axis values represent the median TOTNO_2_ value of each bin shown in panel (a).

For the analyses presented in this section, we excluded TOTNO_2_ measurements where the SZA exceeded 70°, cloud fractions were higher than 15%, and the “main_data_quality_flag” were not equal to zero. The histogram displayed in Figure 3a indicates that a significant proportion of the TEMPO-Pandora data pairs, specifically 55,773 out of 77,958 total samples or 72%, fall within the TOTNO_2_ range of 3 × 10^15^ to 9 × 10^15^ molecules/cm^2^. The maximum TEMPO TOTNO_2_ recorded in the histogram is 8.50 × 10^16^ molecules/cm^2^, however, it excludes bins with TOTNO_2_ values exceeding 45 × 10^15^ molecules/cm^2^ where sample sizes are smaller than 40, as evaluation metrics derived from small sample sizes may lack reliability. Figures 3b–3d illustrate the percentage bias—MABP, RMSEP, and MBP—across different TOTNO_2_ ranges. Generally, the percentage bias (MABP) slightly increases with higher TOTNO_2_ levels, although this increase is not continuous. Specifically, Figure 3b illustrates that the highest MABP, at 34%, occurs within the high TOTNO_2_ range of 42 × 10^15^ to 43.5 × 10^15^ molecules/cm^2^, compared to the lower and medium TOTNO_2_ levels. Conversely, the lowest MABP, at 23.4%, is observed at the lower TOTNO_2_ levels, ranging from 4.5 × 10^15^ to 6 × 10^15^ molecules/cm^2^. Furthermore, Figure 3d shows that TEMPO tends to overestimate TOTNO_2_ at lower NO_2_ levels but underestimates at higher levels, with a notable transition around 12.8 × 10^15^ molecules/cm^2^ where the MBP is nearly zero. The extent of overestimation or underestimation remains moderate, with a maximum overestimation of 16% at TOTNO_2_ ranging from 1.5 × 10^15^ to 3 × 10^15^ molecules/cm^2^ and a maximum underestimation of 31% at TOTNO_2_ ranging from 42 × 10^15^ to 43.5 × 10^15^ molecules/cm^2^. This is an indicator of high capability of TEMPO at measuring TOTNO_2_.

This pattern of initial overestimation shifting to underestimation at higher NO_2_ levels was also observed in previous studies (Ghahremanloo et al., 2024; Lambert et al., 2025; Verhoelst et al., 2021). For instance, Ghahremanloo et al. (2024) validated GEMS Level-2 (v2) TRPNO_2_ against various Pandora stations from 2021 to 2023. The study reported that GEMS tended to overestimate TRPNO_2_ at levels below 15 × 10^15^ molecules/cm^2^ and consistently underestimated above this threshold. The shift from overestimation at lower NO_2_ levels to underestimation at higher levels in Ghahremanloo et al. (2024) highlighted the variability in GEMS’s measurement accuracy across different TRPNO_2_ levels. Moreover, Verhoelst et al. (2021), in validating TROPOMI TOTNO_2_ against Pandora instruments, observed overestimation at low NO_2_ levels and underestimation in high NO_2_ conditions, reinforcing this recurring pattern in satellite-based NO_2_ retrievals.

The discrepancies observed in overestimating or underestimating TOTNO_2_ at various levels may stem from several factors. For instance, all satellite instruments, including TEMPO, have finite spatial resolution, and this can lead to spatial averaging effects. The TEMPO footprint results in a spatially-averaged value representing the mean TOTNO_2_ over a ∼10 km^2^ area. In densely populated urban areas with spatially heterogeneous NO_2_ levels, this spatial averaging effect can lead to underestimations of peak values and overestimations in less polluted suburban areas. Previous studies (Kim et al., 2016; Pinardi et al., 2020), noted that the coarse spatial resolution of instruments like OMI tends to smooth out NO_2_ plumes, resulting in underestimations in urban cores and overestimations in cleaner regions. Pinardi et al. (2020) averaged OMI TRPNO_2_ within varying radii centered on Pandora stations and observed that this spatial averaging had different effects depending on the station’s location. Specifically, the averaging tended to increase TRPNO_2_ levels at rural (clean) sites while decreasing them at urban (polluted) sites. These findings further confirm that the spatial averaging effect in satellite data can lead to overestimation (underestimation) of NO_2_ in rural (urban) regions. Judd et al. (2019) also found that reducing spatial resolution from the finer 250 m scale of the GeoTASO airborne sensor to the coarser scales typical of satellite instruments like TEMPO diminishes the correlation of TRPNO_2_ with ground-based Pandora data. This decline is particularly pronounced in areas with significant NO_2_ spatial variability, attributing to the spatial averaging effect inherent in satellite measurements, which can lead to overestimation in regions of low NO_2_ levels and underestimation in areas with high levels (Judd et al., 2019).

### 4.3. Impact of SZA on TEMPO TOTNO_2_

**Figure 4.**
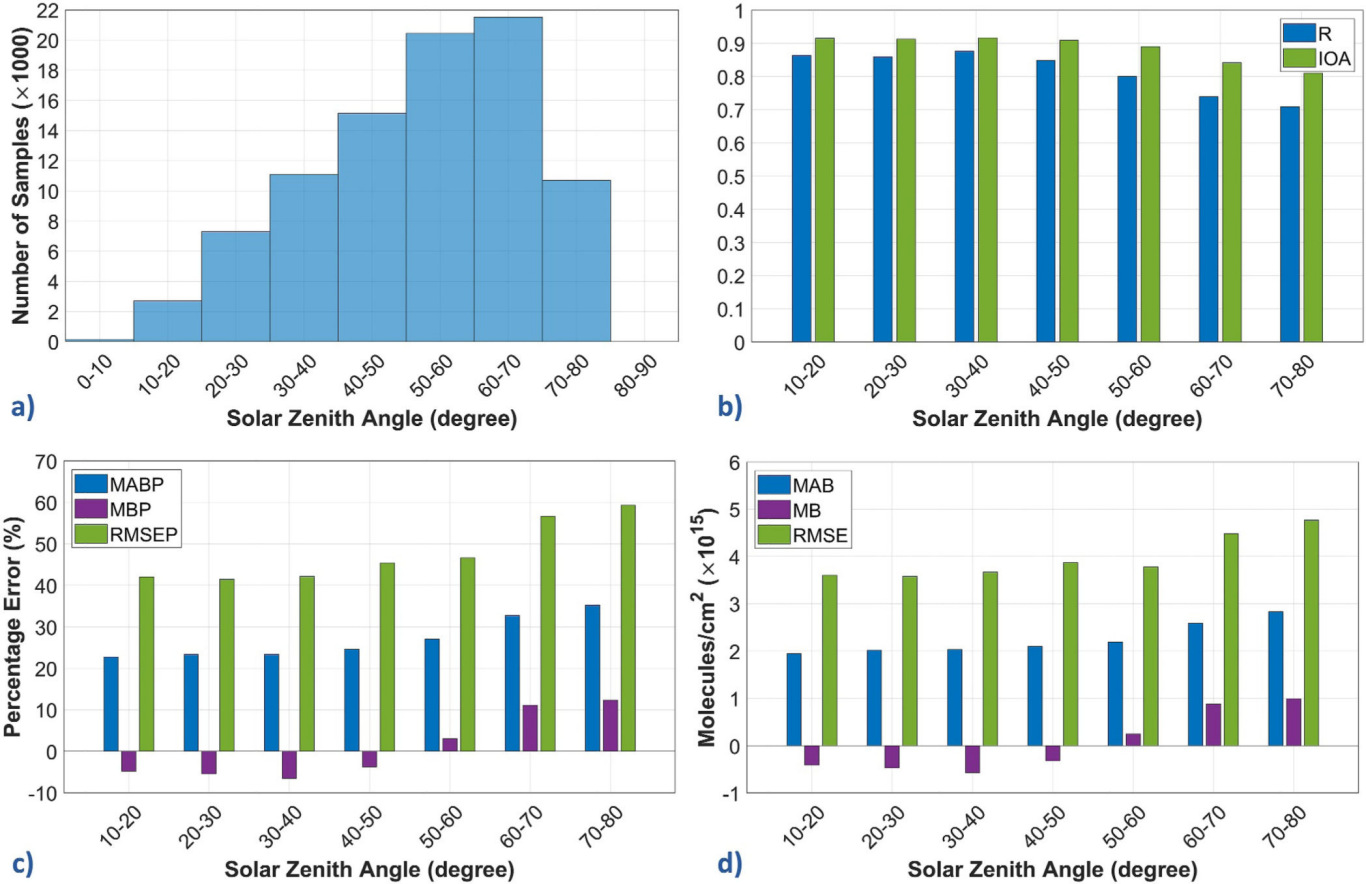
Performance of TEMPO TOTNO_2_ across different Solar zenith angle (SZA) ranges: (a) histogram of SZA ranges for TEMPO-Pandora pairs, (b) correlation coefficient (R) and index of agreement (IOA), (c) percentage bias metrics (MABP, MBP, RMSEP), and (d) absolute bias metrics (MAB, MB, RMSE).

Figure 4 evaluates TEMPO TOTNO_2_ against Pandora TOTNO_2_ in different SZA ranges to show the effect of SZA on the performance of TEMPO TOTNO_2_. For these analyses, we excluded TOTNO_2_ measurements where the cloud fraction exceeded 15%, and the “main_data_quality_flag” were not equal to zero. The histogram of the TEMPO-Pandora pairs in Figure 4a shows a very small number of TEMPO-Pandora samples in SZA range of 0–10° and no samples in the SZA range of 80–90° (TEMPO does retrieve NO_2_ slant column densities at these high SZA, but these observations were removed in our analysis by the application of the quality flag filter). One major reason for the low number of TEMPO–Pandora pairs in the 0–10° SZA range is the limited number of Pandora stations located in the southern part of the TEMPO domain. The highest performance of TEMPO TOTNO_2_ occurs in the SZA range of 10–20° (R: 0.87; IOA: 0.92; MAB: 1.949 × 10^15^ molecules/cm^2^; MABP: 22.7%), where R and IOA are high and bias is lowest. Results show a general decrease in R and IOA and increase in bias as SZA increases from 0° to 80°. Therefore, the lowest performance of TEMPO TOTNO_2_ occurs in the SZA range of 70–80° (R: 0.71; IOA: 0.82; MAB: 2.832 × 10^15^ molecules/cm^2^; MABP: 35.2%). In addition, the underestimation in TEMPO TOTNO_2_ gradually converts to an overestimation with increasing SZA. While TEMPO underestimates TOTNO_2_ by approximately 4%–7% from 10° to 50° SZA, it overestimates TOTNO_2_ by 3%–12% in the SZA range of 50°–80°. Moreover, changes in the SZA from 10° to 40° appear to minimally affect TEMPO’s TOTNO_2_ performance, with the majority of performance degradation occurring as SZA increases from 40° to 80°.

Higher SZA result in the sun being positioned lower on the horizon, which extends the atmospheric path that sunlight must travel. This longer atmospheric path intensifies both scattering and absorption phenomena, posing challenges in obtaining accurate NO_2_ measurements. Several studies (Bracher et al., 2005; Hong et al., 2017) suggest that high SZA may decrease retrieval accuracy due to these geometric considerations. Another factor contributing to discrepancies in TOTNO_2_ performance across different SZA ranges could be the directional nature of Pandora’s viewing geometry. At high SZAs, when the Sun is lower on the horizon, Pandora’s observations—aiming at the Sun in Direct-Sun mode—can be more influenced by NO_2_ levels in the locations along its slanted line of sight. To mitigate this effect, some studies (e.g., Nowlan et al., 2018) have averaged NO_2_ values across pixels located along Pandora’s line of sight before comparing them with airborne or satellite NO_2_ data.

### 4.4. Performance of TEMPO TOTNO_2_ for Different Observation Hours

**Figure 5.**
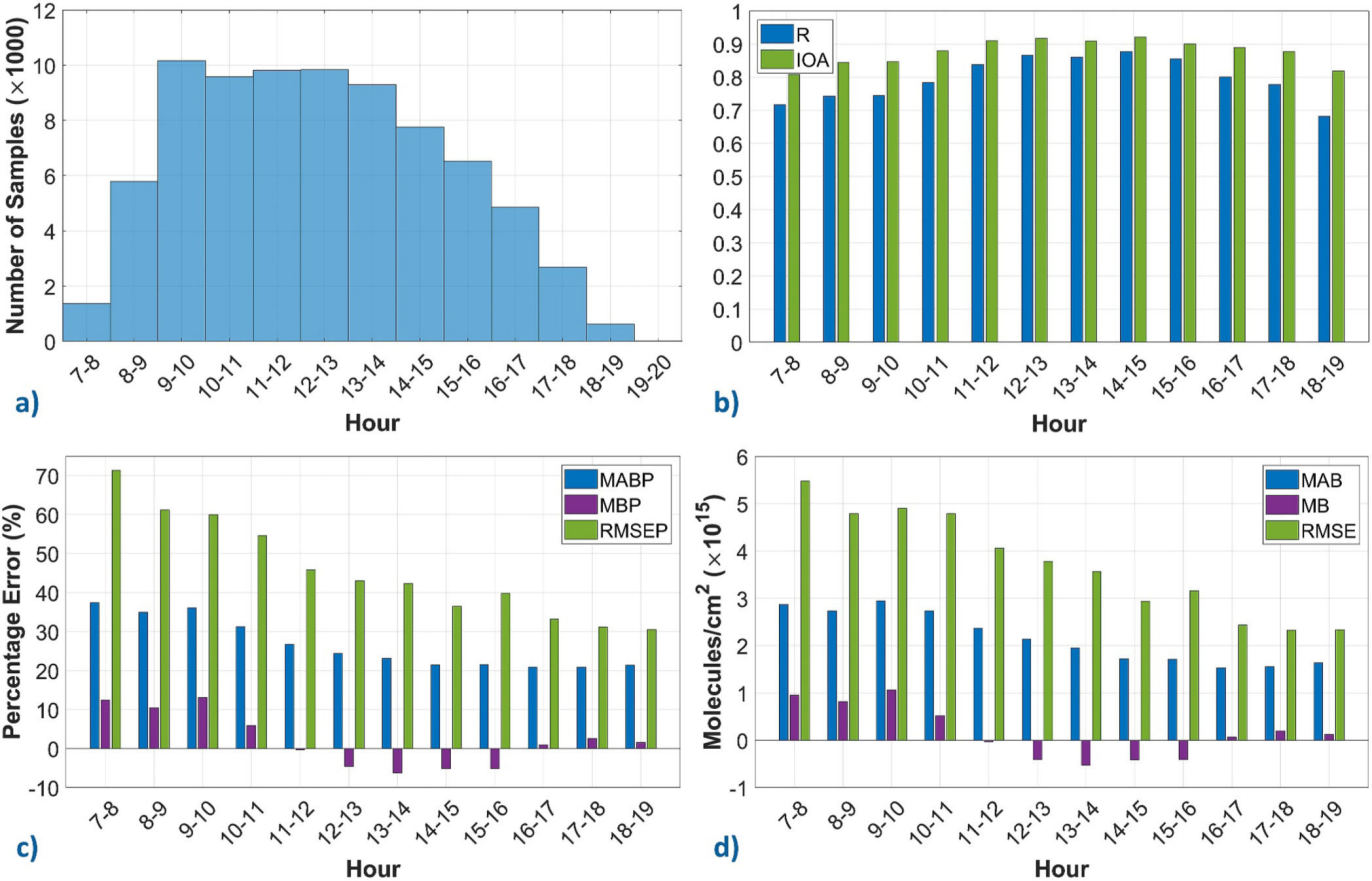
Performance of TEMPO TOTNO_2_ across different observation hours in local time: (a) histogram of observation times (local time) for TEMPO-Pandora pairs, (b) correlation coefficient (R) and index of agreement (IOA), (c) percentage bias metrics (MABP, MBP, RMSEP), and (d) absolute bias metrics (MAB, MB, RMSE).

We examined the influence of observation hours on TEMPO TOTNO_2_ accuracy by converting TEMPO–Pandora pairs observation times from UTC to local time, based on the time zone of each station. We excluded TEMPO measurements with cloud fractions above 15%, SZA greater than 70°, and where the “main_data_quality_flag” were not equal to zero. The histogram in Figure 5a presents the distribution of local observation times for the TEMPO-Pandora pairs from 2 August 2023 to 31 December 2024 in all Pandora stations. As shown in Figure 5a, the shape of histogram is not symmetrical around noon and we have much fewer pairs during early morning (7–8 a.m.) and late afternoon/near sunset (6–8 p.m.) compared to midday. This pattern is primarily driven by the higher concentration of Pandora instruments along the East Coast, where more morning pairs of the TEMPO–Pandora are available, compared to the relative scarcity of Pandora instruments on the West Coast, where more afternoon pairs occur. This disparity could also stem from higher SZA in the morning and late afternoon, complicating the acquisition of reliable measurements due to larger uncertainties in high SZA scenarios. Our quality-control filters applied to TEMPO data further reduce the data set by excluding measurements taken under high SZA situations, further contributing to a reduced number of valid pairs during these periods. Additionally, variations in daylight due to seasonal changes and the geographic locations of Pandora stations affect data availability, with potential full daylight at 7 or 8 p.m. during summer months at certain latitudes, while in winter, it may be dark at these times.

Figure 5 details the accuracy of TOTNO_2_ measurements by TEMPO across different times of the day. It is important to note the exclusion of evaluation metrics for the 7–8 p.m. (19–20) range due to small number (16 samples) of TEMPO-Pandora pairs, whereas metrics for other hours remain reliable. Midday measurements typically show greater accuracy compared to early-morning and near-sunset hours, likely due to lower SZAs that shorten the path of sunlight through the atmosphere and increase sensitivity to the lower layers of the atmosphere (Bracher et al., 2005; Hong et al., 2017). The strongest agreement between TEMPO and Pandora data occurs around midday and early afternoon, demonstrated by higher R (Figure 5b), better IOA (Figure 5b), and moderate to low biases (Figures 5c and 5d). The TEMPO TOTNO_2_ obtained the highest agreement with Pandora at 14 (2:00 p.m.) when R and IOA are 0.88 and 0.92, respectively, and MABP is 21.4%. Although midday and early afternoon hours (e.g., around 11–14) exhibited higher R and IOA, indicator of strong correlation with Pandora data, the lowest RMSEP was observed later in the afternoon. Notably, TOTNO_2_ data measured around 18:00 achieved the smallest RMSEP (30.5%), indicating fewer large deviations from reference Pandora observations. This might be attributed to high PBL height during midday and afternoon hours (e.g., 18:00). The MBP results in Figure 5c also show that TEMPO underestimated TOTNO_2_ during the midday from 11:00 to 15:00, while overestimating it in hours close to the early morning or sunset.

Variations in the performance of TEMPO TOTNO_2_ throughout the day could also be attributed to changes in the PBL height. The diurnal variability of the PBL height and its representation in the GEOS-CF a priori profile significantly affects the accuracy of satellite-derived TOTNO_2_. Since the majority of NO_2_ is emitted at ground level, the daily cycle in PBL height strongly modulates the distribution of NO_2_ within the lower troposphere (Lamsal et al., 2014). In the morning, the PBL is shallow, leading to concentrated NO_2_ near the surface. As solar heating intensifies, the PBL expands, diluting surface NO_2_ concentrations and distributing NO_2_ over higher altitudes. Satellite instruments, which are generally more sensitive to NO_2_ at these higher altitudes, tend to yield more accurate retrievals when the PBL is elevated. Conversely, close to the evening, the PBL height decreases, reconcentrating NO_2_ near the surface and complicating accurate measurements. The retrieval algorithms that convert observed radiances into NO_2_ VCDs rely heavily on accurate modeling of the NO_2_ profile. When the PBL height is underestimated or overestimated in the GEOS-CF a priori profile and thus in the AMF calculation, the retrieved NO_2_ column can be biased. Moreover, errors in the timing of PBL growth within GEOS-CF can disproportionately affect morning TOTNO_2_ measurements, when the PBL is rapidly changing (Yang et al., 2023). This may further help explain the higher retrieval biases observed during morning hours compared to later in the day.

Another key factor contributing to the diurnal variation in TEMPO TOTNO_2_ performance may be biases in modeling the hourly changes in NO_2_ emissions and stratospheric NO_2_ columns within the GEOS-CF a priori NO_2_ profiles. Li et al. (2021) demonstrated that photochemical cycling in the middle atmosphere causes the stratospheric NO_2_ column to increase by approximately 75% from sunrise to late afternoon, followed by a decline overnight. This variation is most pronounced in the lower stratosphere, between 18 and 34 km. If the GEOS-CF a priori profiles do not accurately capture this substantial diurnal variation, it can introduce time-dependent biases in TEMPO TOTNO_2_ retrievals.

### 4.5. Performance of TEMPO TOTNO_2_ at Different Pandora Sites

**Figure 6.**
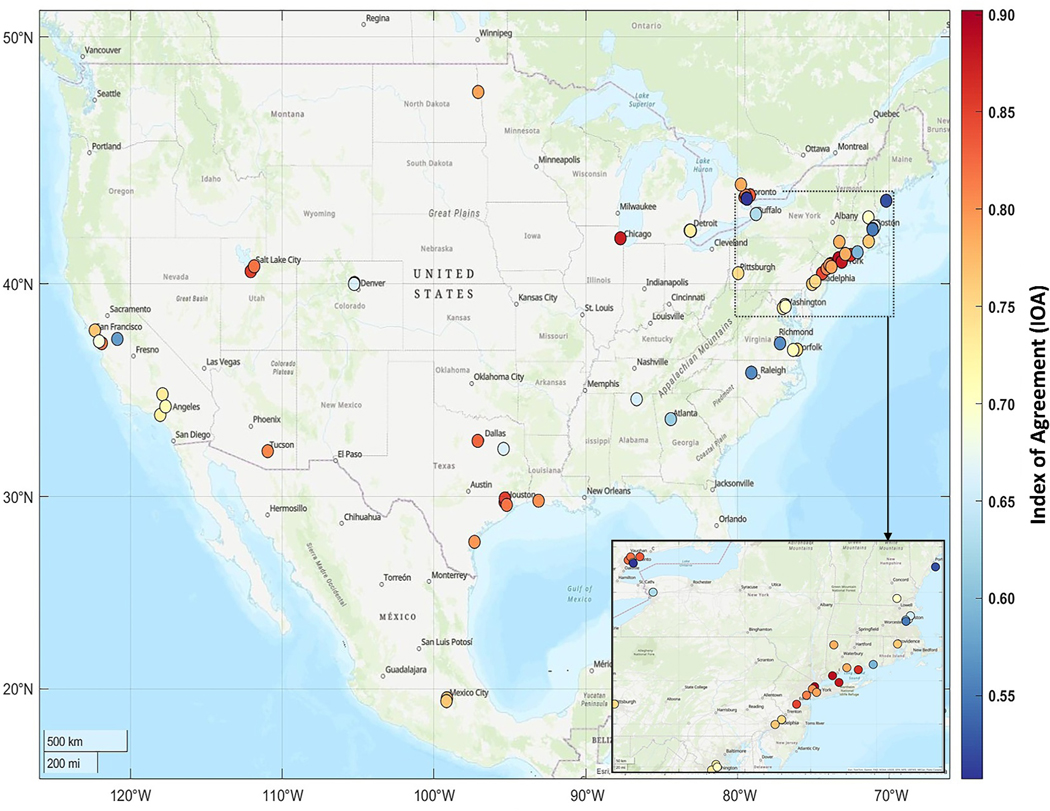
Performance of TEMPO TOTNO_2_ in various Pandora stations in the TEMPO domain. The map shows the values of the index of agreement (IOA) between TEMPO TOTNO_2_ and Pandora observations.

We made separate validations of TEMPO TOTNO_2_ measurements at each of 65 Pandora stations across the United States, Canada, and Mexico from 2 August 2023 to 31 December 2024. Table S2 in Supporting Information S1 lists each station along with computed evaluation metrics demonstrating the measurement accuracy of TEMPO TOTNO_2_. According to this table, the accuracy of TEMPO TOTNO_2_ varied across stations, with R ranging from 0.29 to 0.84, IOA from 0.51 to 0.90, MAB from 6.6 × 10^14^ to 1.00 × 10^16^ molecules/cm^2^, MB from an overestimation of 4.17 × 10^15^ molecules/cm^2^ to an underestimation of − 8.5 × 10^15^ molecules/cm^2^, and RMSE from 9.10 × 10^14^ to 1.31 × 10^16^ molecules/cm^2^. The percentage biases showed MABP ranging from 14.9% to 49.3%, MBP from an overestimation of 43.5% to an underestimation of 29.2%, and RMSEP from 19.2% to 71.1%. In this study, TEMPO TOTNO_2_ demonstrated the highest correlation with Pandora observations at a station in Bronx, New York (R: 0.84; IOA: 0.90), followed by Westport, CT (R: 0.80; IOA: 0.89), and Salt Lake City, UT (R: 0.79; IOA: 0.88). However, these stations did not exhibit the lowest bias for TEMPO TOTNO_2_. The lowest bias was observed at a Pandora station in Grand Forks, ND (MABP: 14.9%), followed by Edwards, CA (MABP: 16.5%), and Arlington, TX (MABP: 17.9%). Conversely, the most significant underestimation occurred at a Pandora station in Boulder, CO (MBP: − 29.2%), while the greatest overestimation was noted at the CN Tower in Toronto, Canada (MBP: 43.5%). Figure 6 also depicts the accuracy (IOA values) of TEMPO TOTNO_2_ at each Pandora station within the TEMPO domain. Spatial maps displaying other evaluation metrics at each station presented in Figures S2–S5 of Supporting Information S1.

Results show significant discrepancies between the accuracy of TEMPO TOTNO_2_ at different Pandora sites. Ghahremanloo et al. (2024) also found comparable variability in the accuracy of GEMS Level-2 (v2) TRPNO_2_ measurements at 18 Pandora stations during the 2021–2023 period, with R values ranging from 0.46 to 0.80, IOA from 0.47 to 0.85, and MABP from 45% to 105%. Similarly, Kim et al. (2023) investigated a smaller set of Pandora stations and noted variations in the accuracy of GEMS TOTNO_2_ against Pandora observations at four stations in South Korea from November 2020 to January 2021 (R: 0.62 to 0.78; RMSE: 3.3 × 10^15^ to 5 × 10^15^ molecules/cm^2^). They also reported changes in TROPOMI’s accuracy in the same stations, with R ranging from 0.58 to 0.74 and RMSE from 3.8 × 10^15^ to 7 × 10^15^ molecules/cm^2^. Furthermore, Li et al. (2023) identified varying accuracies of GEMS TRPNO_2_ when compared with four commercial MAX-DOAS instruments across China, noting R values from 0.69 to 0.92.

One primary factor contributing to the observed discrepancies in the accuracy of TEMPO TOTNO_2_ is the altitude of the Pandora stations relative to the ground. Sometimes positioned at elevated locations such as the rooftops of tall buildings to maintain unobstructed sunlight access, certain Pandora instruments may not detect significant portions of NO_2_ columns below their altitude. However, TEMPO can detect these near-surface NO_2_ levels as it measures the entire ground-based Pandora observations. This observation is critically important as NO_2_ concentrations are higher near the Earth’s surface, and progressively decrease with altitude (Liu & Wang, 2024; Merlaud et al., 2011).

Notably, the lowest accuracy of TEMPO TOTNO_2_ in our study (R: 0.36; IOA: 0.51; MABP: 49.33%; MBP: 43.54%) was observed at the Pandora station installed approximately 330 m above ground level on the CN Tower in Toronto, Canada. Here, TEMPO significantly overestimated TOTNO_2_ compared to the Pandora instrument, due to the Pandora instrument’s inability to detect NO_2_ levels beneath its altitude, whereas TEMPO’s measurements from space capture near-surface NO_2_ levels. Interestingly, the next two Pandora stations exhibiting the highest bias (MABP) in TEMPO TOTNO_2_ measurements are also located at relatively high altitudes: one in Manhattan, New York, with an MABP of 48.7% at 32 m above ground, and another in New London, Connecticut, with an MABP of 38.7% at 20 m above ground. Like the Pandora station on the CN Tower in Toronto, TEMPO also overestimated TOTNO_2_ at Pandora stations located in these two stations: Manhattan, New York (MBP: 35.6%) and New London, Connecticut (MBP: 28.9%).

The correlation between the altitude of Pandora instruments relative to the ground and the MABP of TEMPO TOTNO_2_ across these stations is *R* = 0.50. This positive relationship indicates that an increase in the altitude of Pandora stations relative to the ground is associated with higher bias of TEMPO TOTNO_2_ compared to Pandora observations. Additionally, Table S2 in Supporting Information S1 shows that the altitude of Pandora stations exerts a stronger influence on the bias (i.e., MABP) of TEMPO TOTNO_2_ relative to Pandora than on the correlation between TEMPO and Pandora. This is further supported by the correlation between the altitude of all Pandora stations and the R of TEMPO TOTNO_2_ at these stations, which is lower at *R* = − 0.40. These analyses show that scientists must exercise caution when choosing the locations of Pandora instruments with respect to the instrument’s altitude relative to ground. Previous studies have also shown that Pandora instruments can miss a notable portion of NO_2_ when installed at elevated heights above the ground. Nowlan et al. (2016) examined NO_2_ column measurements from two Pandora instruments installed at different altitudes—70 m and 7 m—at the Moody Tower site in Houston, Texas. They found that the NO_2_ column observed at the higher altitude (70 m) was nearly 68% lower than that measured near the surface (7 m), indicating a substantial portion of the NO_2_ resides close to the ground. This highlights the strong vertical gradient of NO_2_ in polluted urban environments, where elevated installations may miss a significant fraction of the total column.

Additionally, other factors may contribute to the varying accuracy of TEMPO TOTNO_2_ measurements at different stations. One such factor is population density, which reflects human activity in the vicinity of the Pandora stations. We derived population density data for the areas surrounding the Pandora stations from the gridded population density data set at a 1 km spatial resolution provided by the NASA Socioeconomic Data and Applications Center (Center for International Earth Science Information Network, 2016). Our analysis revealed a positive correlation between the MAB of TEMPO TOTNO_2_ in all stations and population density in these stations, with an R of 0.55. Similarly, an R of 0.43 was observed for the percentage bias (MABP). This indicates that the bias in TEMPO TOTNO_2_ measurements compared to Pandora observations increases with the population density at the locations of the Pandora stations. Among several potential reasons, this trend of increasing bias in TEMPO TOTNO_2_ with population density might be attributed to the fact that areas with higher population densities typically have greater aerosol content, which can compromise the accuracy of satellite NO_2_ retrievals. Aerosols interfere with these measurements by scattering and absorbing light, posing significant challenges for satellites that depend on spectrometric data obtained from the top of the atmosphere (Liu et al., 2024). Moreover, significant spatial variability of NO_2_ in urban regions—especially in areas with higher human activity—may further contribute to the lower agreement between TEMPO TOTNO_2_ and Pandora observations in dense urban cores.

Spatial variability in NO_2_ becomes particularly important when comparing satellite-derived TOTNO_2_ with Pandora observations, due to the directional nature of Pandora’s viewing geometry. Nowlan et al. (2018) compared NO_2_ column measurements from the GEOstationary Coastal and Air Pollution Events (GEO-CAPE) Airborne Simulator (GCAS) with Pandora observations in Houston, Texas. In one of their study stations, the GCAS consistently recorded high NO_2_ columns within the pixel containing the Pandora instrument, with often much higher NO_2_ levels to the north and cleaner air to the south of the GCAS pixel. Because Pandora always points directly at the Sun during Direct-Sun observations, it was consistently oriented southward—toward the cleaner air mass—underestimating GCAS NO_2_ column. To account for this mismatch in sampling geometry, the authors averaged GCAS NO_2_ values in the pixels located along Pandora’s line of sight. This adjustment reduced the GCAS–Pandora bias by approximately 20%, underscoring the critical role of NO_2_ spatial heterogeneity in satellite–Pandora NO_2_ comparisons, including those involving TEMPO. In addition to other reasons, the GEOSCF model currently uses the HTAPv2 2010 emission inventory that does not reflect emission reductions. This may lead to overestimation of near-surface NO_2_ and underestimation of the AMF. An underestimation in the AMF could also explain an increasing bias in the TOTNO_2_.

### 4.6. Comparing Accuracy: TEMPO Versus TROPOMI Versus OMI

We extracted TEMPO TOTNO_2_ data at the overpass time of the TROPOMI and OMI and aligned it with data from Pandora stations. This method ensured a consistent basis for comparison, enabling a precise evaluation of each satellite instrument’s TOTNO_2_ measurements against the ground-based Pandora observations. The equatorial overpass time for both TROPOMI and OMI occurs around 13:30 local time, hence observations for TEMPO-TROPOMI-OMI-Pandora pairs were collected between 13:30 and 14:00 local time. Table 1 shows the key specifications of the satellite instruments (i.e., TEMPO, TROPOMI, and OMI), such as the orbit, spatial resolution, a-priori vertical NO_2_ profile, and so on.

Table 2 presents the validation results of TOTNO_2_ measured by TEMPO, TROPOMI, and OMI against Pandora observations. This table shows two separate sets of comparative validations: (a) comparing TEMPO and TROPOMI with Pandora observations, and (b) including all three instruments in the comparison. The strategy for this two-tier validation approach was to maximize sample sizes for robust evaluation, particularly for TEMPO-TROPOMI-Pandora comparisons (Table 2a). A smaller sample size in Table 2b resulted from OMI’s sensor degradation and row anomalies, reducing valid data points. Another limiting factor was that the inclusion of TEMPO-TROPOMI-OMI-Pandora pairs in our analysis was contingent upon the availability of concurrent measurements from all three sensors. This requirement significantly reduced the number of samples available for Table 2b.

**Figure 7.**
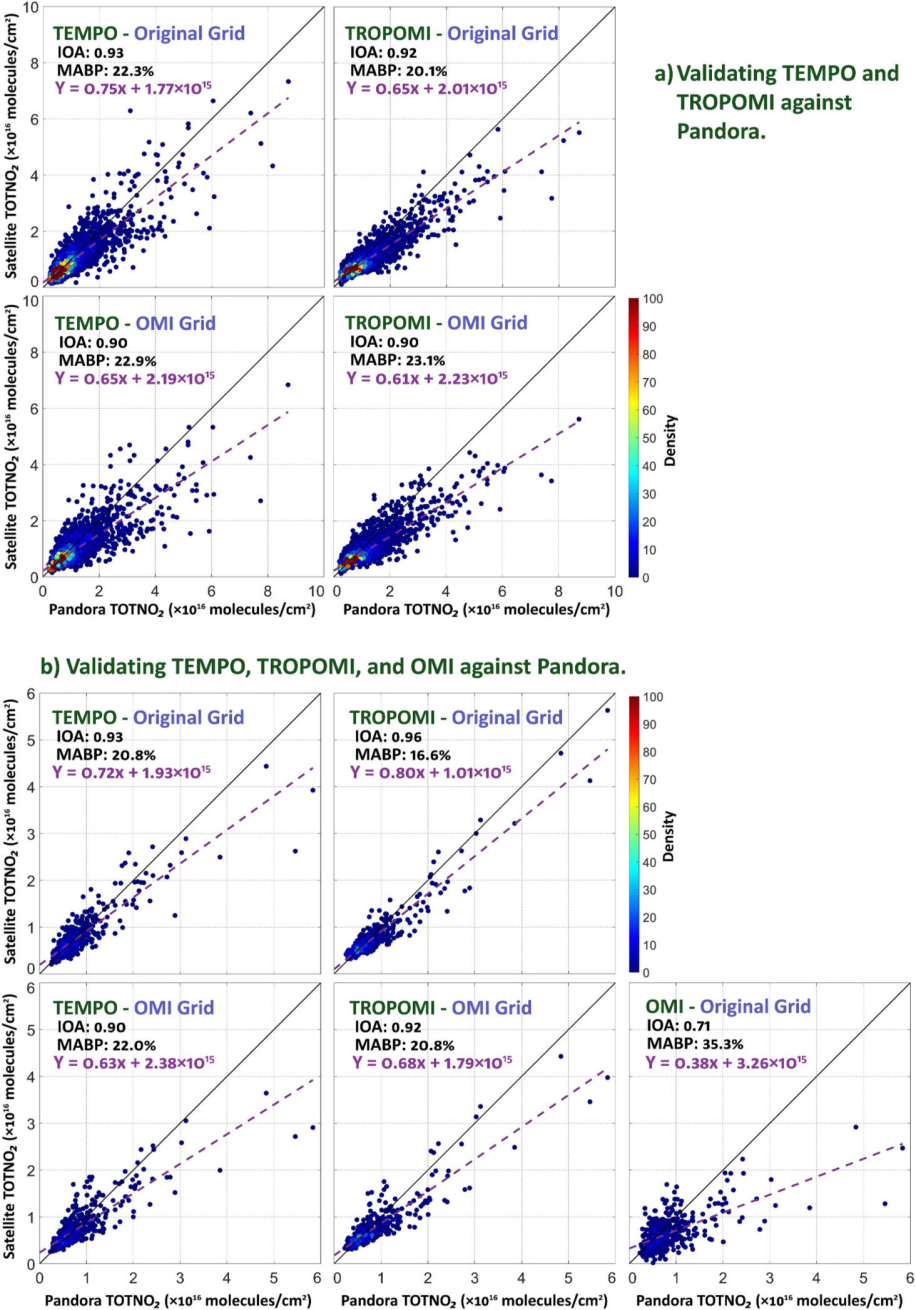
Validation of TOTNO_2_ from TEMPO, TROPOMI, and Ozone Monitoring Instrument (OMI) against Pandora data at TROPOMI and OMI overpass times. The figure is divided into panels (a, b) to optimize sample size and ensure a robust evaluation, particularly in panel (a). Validation is shown using both original and regridded (resampled to OMI grid) satellite data to assess the impact of spatial resolution on TOTNO2 accuracy.

To evaluate the impact of spatial resolution on the accuracy of TOTNO_2_ retrievals from each satellite instrument, we resampled TEMPO and TROPOMI data to match the coarser OMI pixel size, with a spatial resolution of 13 × 24 km^2^. Consequently, each section of (a) and (b) in Table 2 presents results based on both the original instrument grids and the regridded data at OMI resolution. Figure 7 visualizes the same results shown in Table 2, using two sets of comparative validations: (a) compares TEMPO and TROPOMI against Pandora observations, while (b) includes OMI in the comparison.

In regard to the satellite TOTNO_2_ in their original grids, Figures 7a and Table 2a indicate that TEMPO achieved a higher IOA of 0.93 compared to TROPOMI’s 0.92 and exhibited a lower MB of − 0.270 × 10^15^ molecules/cm^2^ against − 0.863 × 10^15^ molecules/cm^2^ for TROPOMI, when validated against Pandora observations. TEMPO showed a slight underestimation of TOTNO_2_ by MBP = − 3.3%, while TROPOMI’s underestimation reached MBP = − 10.5%. However, TROPOMI’s TOTNO_2_ measurements correlate more strongly with Pandora’s TOTNO_2_ (R: 0.91) compared to TEMPO (R: 0.87). Moreover, the absolute and percentage bias of TOTNO_2_ measured by TROPOMI (MAB: 1.662 × 10^15^ molecules/cm^2^; MABP: 20.1%) are slightly lower than those observed in TEMPO (MAB: 1.838 × 10^15^ molecules/cm^2^; MABP: 22.3%). Figure 7b and Table 2b further demonstrate these comparisons including OMI, with notably fewer samples (529) compared to the extensive data set used in Figure 7a (5,120 samples). Results underscore the high accuracy of both TEMPO (R: 0.88; IOA: 0.93; MABP: 20.8%) and TROPOMI (R: 0.94; IOA: 0.96; MABP: 16.6%), while, OMI lags with R, IOA, and MABP of 0.62%, 0.71%, and 35.3%. The lower performance of OMI TOTNO_2_ can be attributed to the coarse spatial resolution, sensor degradation, and row anomaly (Torres et al., 2018). It should be highlighted that the spatial resolution of OMI, at 13 × 24 km^2^, is significantly coarser than that of TEMPO (2 × 4.75 km^2^ in Level-2) and TROPOMI (3.5 × 5.5 km^2^). Consequently, the relatively modest correlation between OMI TOTNO_2_ measurements and Pandora observations can be partially attributed to OMI’s larger spatial resolution, rather than an inherent inaccuracy in the instrument. This distinction is critical for interpreting comparative data accuracy assessments.

To assess the impact of spatial resolution on the accuracy of TOTNO_2_ retrievals, TEMPO and TROPOMI data were resampled to the coarser OMI pixel size (13 × 24 km^2^). According to the results in Table 2a and Figure 7a, this resampling led in a slight decrease in the accuracy of TOTNO_2_ estimates from both instruments. For TEMPO, the values of R, IOA, and MABP decreased from 0.87%, 0.93%, and 22.3% in the original grid to 0.84%, 0.90%, and 22.9% in the regridded data, respectively. Similarly, TROPOMI’s performance dropped from R = 0.91, IOA = 0.92, and MABP = 20.1% in the original grid to R = 0.88, IOA = 0.90, and MABP = 23.1% after regridding. Despite this decline, the results in Table 2b and Figure 7b demonstrate that the regridded TOTNO_2_ data from TEMPO (R: 0.85; IOA: 0.90; MABP: 22.0%) and TROPOMI (R: 0.90; IOA: 0.92; MABP: 20.8%) at the OMI spatial resolution still significantly outperform the accuracy of OMI TOTNO_2_ (R: 0.62; IOA: 0.71; MABP: 35.3%).

The observed discrepancies in the accuracy of TOTNO_2_ measurements across the TEMPO, TROPOMI, and OMI satellites can also be traced to a variety of other sources, including the unique retrieval algorithms employed by each satellite, their specific viewing geometries, and distinct instrument characteristics such as spectral resolution, radiometric sensitivity, and calibration methods (Ghahremanloo et al., 2024; Zhang et al., 2018). In their research, Ghahremanloo et al. (2024) also leveraged Pandora observations to validate Level-2 (v2) TRPNO_2_ measurements from GEMS and TROPOMI during TROPOMI overpass times from 2021 to 2023. Their findings revealed that GEMS achieved satisfactory results in TRPNO_2_ measurements at TROPOMI’s overpass times, with R, IOA, MAB, and MABP values of 0.71, 0.78, 6.822 × 10^15^ molecules/cm^2^, and 72%, respectively. However, TROPOMI exhibited superior accuracy, reporting R, IOA, MAB, and MABP values of 0.81, 0.89, 3.268 × 10^15^ molecules/cm^2^, and 35%, respectively. This notable discrepancy highlighted TROPOMI’s enhanced capability in TRPNO_2_ monitoring, with its MABP approximately half that of GEMS. Additionally, GEMS Level-2 (v2) TRPNO_2_ showed a significant overestimation of TRPNO_2_ levels by 52%, whereas measurements from TROPOMI indicated a more conservative underestimation of 9%. In comparison to these findings, our study suggests that TEMPO may provide better accuracy in NO_2_ measurement compared to GEMS Level-2 (v2) data, despite the challenges in conducting a direct comparison between the two sensors due to their fixed positions over different geographical regions.

## 5. Conclusion

TEMPO is the first geostationary satellite instrument that continuously monitors several criteria air pollutants, including NO_2_, across North America. Launched on 7 April 2023, TEMPO achieved first light on 2 August 2023. This study provides a comprehensive analysis of bias in TEMPO TOTNO_2_ using TOTNO_2_ observations from 65 Pandora spectrometers in the United States, Canada, and Mexico, spanning 2 August 2023, through 31 December 2024. Various scenarios were examined to evaluate the performance of TEMPO TOTNO_2_ under different conditions. TEMPO showed strong accuracy at cloud filtering threshold of 5%: R of 0.86, IOA of 0.91, MAB of 1.423 × 10^15^ molecules/cm^2^, and an MABP of 23.1%, corresponding to an underestimation of 12% against Pandora observations. Accuracy decreased when pixels with greater cloud cover are included. Analysis of TEMPO TOTNO_2_ bias in different cloud fraction ranges showed a 13% underestimation at 0%–5% cloud fraction shifts to an 80% overestimation at 95%–100%. Impact of SZA on TEMPO TOTNO_2_ performance was also investigated. An SZA of 10–20° yielded the highest accuracy (R: 0.87; IOA: 0.92; MAB: 1.949 × 10^15^ molecules/cm^2^; MABP: 22.7%), whereas SZA of 70–80° yielded the lowest (R: 0.71; IOA: 0.82; MAB: 2.833 × 10^15^ molecules/cm^2^; MABP: 35.2%). Consequently, early-morning or near-sunset observations were less accurate than midday. When comparing observation hours, the best agreement (R: 0.88, IOA: 0.92, MABP: 21.4%) of TEMPO TOTNO_2_ with Pandora occurred around 14:00 local time, and the smallest RMSEP (30.5%) in TEMPO TOTNO_2_ occurred around 18:00 local time, indicating fewer large deviations from reference Pandora observations. In general, TEMPO tended to overestimate TOTNO_2_ during early morning and nearsunset, while underestimating it during midday. In addition to SZA, errors in the timing of PBL growth within GEOS-CF can also disproportionately affect morning TOTNO_2_ measurements, when the PBL is rapidly changing. This may further help explain the higher retrieval biases observed during morning hours compared to later in the day.

Typically, TEMPO tended to overestimate Pandora TOTNO_2_ at lower NO_2_ levels and underestimate at higher levels, crossing zero MB near 12.8 × 10^15^ molecules/cm^2^. Maximum overestimation reached MBP = 16% at very low (1.5 × 10^15^ to 3 × 10^15^ molecules/cm^2^) TOTNO_2_ levels, while the maximum underestimation occurred at high (42 × 10^15^ to 43.5 × 10^15^ molecules/cm^2^) NO_2_ levels at 31%. Moreover, percentage bias (i.e., MABP) increased with higher TOTNO_2_ levels. Specifically, the highest MABP, at 34%, occurred within the high TOTNO_2_ range of 42 × 10^15^ to 43.5 × 10^15^ molecules/cm^2^, compared to the lower and medium TOTNO_2_ levels. Station-to-station performance also varied significantly, with R ranging from 0.29 to 0.84, IOA from 0.51 to 0.90, and MABP from 14.9% to 49.3%. Pandora stations installed at higher altitudes relative to ground (such as tall buildings) showed weaker agreement with TEMPO, as Pandora does not measure NO_2_ below the instrument’s altitude, while TEMPO retrieves the entire vertical column. The highest bias in TEMPO TOTNO_2_ in this study was observed at a Pandora station installed ∼330 m above ground level on the CN Tower in Toronto, Canada (R: 0.36; IOA: 0.51; MABP: 49.33%; MBP: 43.54%). In contrast, another Pandora site (University of Toronto’s St. George campus; altitude: 61 m), only 2 km away from the CN Tower, had better agreement with TEMPO (R: 0.56; IOA: 0.72; MABP: 28.6%; MBP: 6.1%), further highlighting the impact of altitude on the agreement between TEMPO and Pandora. Finally, validation of TEMPO TOTNO_2_ at the overpass times of TROPOMI and OMI showed that TEMPO’s performance (R: 0.88, IOA: 0.93, MABP: 20.8%) closely aligns with that of TROPOMI (R: 0.94, IOA: 0.96, MABP: 16.6%) and substantially exceeds that of OMI (R: 0.62, IOA: 0.71, MABP: 35.3%). To assess the impact of spatial resolution on the accuracy of TOTNO_2_ retrievals from each satellite instrument, we resampled TEMPO and TROPOMI data to match OMI’s coarser spatial resolution of 13 × 24 km^2^. The comparative validation at this uniform resolution revealed a modest reduction in accuracy for TEMPO (R: 0.85, IOA: 0.90, MABP: 22.0%) and TROPOMI (R: 0.90, IOA: 0.92, MABP: 20.8%), yet both still significantly outperformed OMI (R: 0.62, IOA: 0.71, MABP: 35.3%).

## Supplementary Material

Supplement1

Supporting Information may be found in the online version of this article.

## Figures and Tables

**Table 1:** Key Specifications of the Satellite Instruments TEMPO, TROPOMI, and Ozone Monitoring Instrument, Whose TOTNO_2_ Measurements Were Validated Against Ground-Based Pandora Observations

	TEMPO (Level-3 v3)	TROPOMI (Level-2 v2)	OMI (Level-2 v4)

Orbit and Data Frequency and Spatial Resolution	Geostationary, hourly (daylight) 2 × 4.75 km at FOR center	LEO Sun-synchronous, daily 3.5 × 5.5 km at nadir	LEO Sun-synchronous, daily 13 × 24 km at nadir
A-Priori Vertical NO_2_ Profile	3D profiles from the GEOS-CF (0.25° × 0.25°, 72 layers)	Daily profiles from the TM5-MP model (1° × 1°, 34 layers)	Monthly-mean NO_2_ profiles from the NASA GMI (1.25° × 1°, 72 layers)
Slant Column Density (SCD) Fitting Window	Direct fitting of radiances in window 405-465 nm	Differential optical absorption spectroscopy (DOAS) window 405–465 nm	Differential optical absorption spectroscopy (DOAS) window 402–465 nm
Surface Albedo Input Used in AMF	Geometry-dependent LER (GLER); Climatology: MODIS BRDF (land); Cox-Munk approximation (water)	Directionally-dependent LER (DLER); Climatology: From OMI	Geometry-dependent LER (GLER); Climatology: MODIS BRDF (land); Cox-Munk approximation (water)
Cloud Correction Approach	TEMPO O_2_-O_2_ cloud algorithm provides cloud fraction/pressure	FRESCO-S O_2_-A-band cloud product provides cloud fraction/pressure	OMI O_2_-O_2_ cloud product provides cloud fraction/pressure

**Table 2:** Validation Results of TOTNO_2_ Measurements From Satellite Instruments

Grid	Instrument	R	IOA	MAB (%)	MB (%)	RMSE (%)	# Obs.

*(a) Validating TOTNO*_*2*_ *from TEMPO and TROPOMI against Pandora*							
Original	TEMPO	0.87	0.93	1.838 (22.3)	−0.270 (−3.30)	3.191 (38.6)	5,120
	TROPOMI	0.91	0.92	1.662 (20.1)	−0.863 (−10.5)	2.972 (36.0)	5,120
OMI	TEMPO	0.84	0.90	1.878 (22.9)	−0.679 (−8.30)	3.512 (42.8)	5,120
	TROPOMI	0.88	0.90	1.897 (23.1)	−0.978 (−11.9)	3.350 (40.8)	5,120
*(b) Validating TOTNO*_*2*_ *from TEMPO, TROPOMI, and OMI against Pandora*							
Original	TEMPO	0.88	0.93	1.634 (20.8)	−0.255 (−3.2)	2.683 (34.2)	529
	TROPOMI	0.94	0.96	1.305 (16.6)	−0.527 (−6.7)	2.019 (25.7)	529
	OMI	0.62	0.71	2.768 (35.3)	−1.537 (−19.6)	4.659 (59.4)	529
OMI	TEMPO	0.85	0.90	1.724 (22.0)	−0.477 (−6.1)	3.065 (39.2)	529
	TROPOMI	0.90	0.92	1.628 (20.8)	−0.676 (−8.7)	2.672 (34.2)	529
	OMI	0.62	0.71	2.768 (35.3)	−1.537 (−19.6)	4.659 (59.4)	529

*Note.* (a) Comparison of TEMPO and TROPOMI with Pandora observations; (b) Comparison of TEMPO, TROPOMI, and OMI with Pandora observations. Each section of (a, b) includes results using the instruments’ original grids and data regridded to the OMI pixel size, to assess the impact of spatial resolution on TOTNO_2_ accuracy comparisons. The MAB, MB, and RMSE are in the unit of 10^15^ molecules/cm^2^. % represents the percentage bias, which is MAB, MB, or RMSE divided by the mean Pandora TOTNO_2_. #Obs. refers to the sample size.

## Data Availability

TEMPO TOTNO_2_ data can be obtained from the NASA Langley Atmospheric Science Data Center at TEMPO_NO2_L3_V03: NASA/LARC/SD/ASDC (2024); Pandora TOTNO_2_ data are available for download at the web portal of the Pandonia Global Network (PGN) (Pandonia Global Network, 2025); The TOTNO_2_ data from TROPOMI and OMI instruments can be collected from the NASA Goddard Earth Sciences Data and Information Services Center (GES-DISC), available at (Copernicus Sentinel data, 2021) and (Krotkov et al., 2019); Population density data are available at the NASA Socioeconomic Data and Applications Center (Center for International Earth Science Information Network, 2016).

## Key Points:

- A comprehensive validation of TEMPO total column density of NO_2_ (TOTNO_2_) (Level-3 v3) was performed using data from 65 Pandora stations
- TEMPO showed high accuracy at measuring TOTNO_2_ (R: 0.86, IOA: 0.91, MABP: 23.1%, underestimating TOTNO_2_ relative to Pandora by 12%)
- TEMPO TOTNO_2_ showed lower agreement with high-altitude (relative to ground) Pandora instruments

## References

[R1] Alwarda R, Bognar K, Zhao X, Fioletov V, Davies J, Lee SC, (2025). Retrieval of NO2 profiles from 3 years of Pandora MAX-DOAS measurements in Toronto, Canada. Atmospheric Measurement Techniques, 18(11), 2397–2423. 10.5194/amt-18-2397-2025

[R2] Boersma KF, Eskes HJ, Dirksen RJ, Van Der A RJ, Veefkind JP, Stammes P, (2011). An improved tropospheric NO2 column retrieval algorithm for the Ozone Monitoring Instrument. Atmospheric Measurement Techniques, 4(9), 1905–1928. 10.5194/amt-4-1905-2011

[R3] Bovensmann H, Burrows JP, Buchwitz M, Frerick J, Noel S, Rozanov VV, (1999). SCIAMACHY: Mission objectives and measurement modes. Journal of the Atmospheric Sciences, 56(2), 127–150. 10.1175/1520-0469(1999)056<0127:smoamm>2.0.co;2

[R4] Bracher A, Sinnhuber M, Rozanov A, & Burrows JP (2005). Using a photochemical model for the validation of NO2 satellite measurements at different solar zenith angles. Atmospheric Chemistry and Physics, 5(2), 393–408. 10.5194/acp-5-393-2005

[R5] Burrows JP, Weber M, Buchwitz M, Rozanov V, Ladstätter-Weißenmayer A, Richter A, (1999). The global ozone monitoring experiment (GOME): Mission concept and first scientific results. JournaloftheAtmosphericSciences, 56(2), 151–175. 10.1175/1520-0469(1999)056<0151:tgomeg>2.0.co;2

[R6] Cede A (2024). Blick Software Suite manual (Version 1.8-5). Retrieved from https://www.pandonia-global-network.org/wp-content/uploads/2024/08/BlickSoftwareSuite_Manual_v1-8-5.pdf

[R7] Cede A, Tiefengraber M, Gebetsberger M, & Spinei E (2025). Pandonia Global Network data products readme document (Version 1.8-10) [PDF]. Retrieved from https://publications.pandonia-global-network.org/manuals/PGN_DataProducts_Readme.pdf

[R8] Center for International Earth Science Information Network - CIESIN - Columbia University. (2016). Gridded population of the world, version 4 (GPWv4): Population density. Palisades, NY: ESDIS [Dataset]. https://www.earthdata.nasa.gov/data/catalog/esdis-ciesin-sedac-gpwv4-popdens-4.0

[R9] Chiusolo M, Cadum E, Stafoggia M, Galassi C, Berti G, Faustini A, & EpiAir Collaborative Group. (2011). Short-term effects of nitrogen dioxide on mortality and susceptibility factors in 10 Italian cities: The EpiAir study. Environmental Health Perspectives, 119(9), 1233–1238. 10.1289/ehp.1002904 21586369 PMC3230391

[R10] Copernicus Sentinel data processed by ESA, Koninklijk Nederlands Meteorologisch Instituut (KNMI). (2021). Sentinel-5P TROPOMI tropospheric NO2 1-Orbit L2 5.5 km x 3.5 km, Greenbelt, MD, USA [Dataset]. GoddardEarthSciences Dataand Information Services Center (GES DISC). 10.5270/S5P-9bnp8q8

[R11] Edwards DP, Emmons LK, Hauglustaine DA, Chu DA, Gille JC, Kaufman YJ, (2004). Observations of carbon monoxide and aerosols from the Terra satellite: Northern Hemisphere variability. Journal of Geophysical Research, 109(D24). 10.1029/2004jd004727

[R12] Fischer PH, Marra M, Ameling CB, Hoek G, Beelen R, de Hoogh K, (2015). Air pollution and mortality in seven million adults: The Dutch Environmental Longitudinal Study (DUELS). Environmental Health Perspectives, 123(7), 697–704. 10.1289/ehp.1408254 25760672 PMC4492265

[R13] Flynn L, Long C, Wu X, Evans R, Beck CT, Petropavlovskikh I, (2014). Performance of the ozone mapping and profiler suite (OMPS) products. Journal of Geophysical Resea*rch*: Atmospheres, 119(10), 6181–6195. 10.1002/2013jd020467

[R14] Frieß U, Beirle S, Alvarado Bonilla L, Bösch T, Friedrich MM, Hendrick F, (2019). Intercomparison of MAX-DOAS vertical profile retrieval algorithms: Studies using synthetic data. Atmospheric Measurement Techniques, 12(4), 2155–2181. 10.5194/amt-12-2155-2019

[R15] Geddes JA, Martin RV, Bucsela EJ, McLinden CA, & Cunningham DJ (2018). Stratosphere–troposphere separation of nitrogen dioxide columns from the TEMPO geostationary satellite instrument. Atmospheric Measurement Techniques, 11(11), 6271–6287. 10.5194/amt-11-6271-2018

[R16] Geddes JA, Murphy JG, O’Brien JM, & Celarier EA (2012). Biases in long-term NO2 averages inferred from satellite observations due to cloud selection criteria. Remote Sensing of Environment, 124, 210–216. 10.1016/j.rse.2012.05.008

[R17] Ghahremanloo M, Choi Y, Sayeed A, Salman AK, Pan S, & Amani M (2021c). Estimating daily high-resolution PM2. 5 concentrations over Texas: Machine Learning approach. Atmospheric Environment, 247, 118209. 10.1016/j.atmosenv.2021.118209

[R18] Ghahremanloo M, Choi Y, & Singh D (2024). Deep learning bias correction of GEMS tropospheric NO2: A comparative validation of NO2 from GEMS and TROPOMI using Pandora observations. Environment International, 190, 108818. 10.1016/j.envint.2024.108818 38878653

[R19] Ghahremanloo M, Lops Y, Choi Y, & Mousavinezhad S (2021a). Impact of the COVID-19 outbreak on air pollution levels in East Asia. Science of the Total Environment, 754, 142226. 10.1016/j.scitotenv.2020.142226 33254896 PMC7476443

[R20] Ghahremanloo M, Lops Y, Choi Y, Mousavinezhad S, & Jung J (2023). A coupled deep learning model for estimating surface NO2 levels from remote sensing data: 15-year study over the contiguous United States. Journal of Geophysical Research: Atmospheres, 128(2), e2022JD037010. 10.1029/2022jd037010

[R21] Ghahremanloo M, Lops Y, Choi Y, & Yeganeh B (2021b). Deep learning estimation of daily ground-level NO2 concentrations from remote sensing data. Journal of Geophysical Research: Atmospheres, 126(21), e2021JD034925. 10.1029/2021jd034925

[R22] Goldberg DL, Nawaz MO, Lyu C, He J, Carlton AG, Kondragunta S, & Anenberg SC (2025). NO2 concentration differences under clear versus cloudy skies and implications for applications of satellite measurements. EGUsphere, 2025, 1–23.

[R23] González Abad G, Nowlan C, Wang H, Chong H, Houck J, Liu X, & Chance K (2024). TEMPO trace gas and cloud level 2 and 3 data products: User guide. Center for Astrophysics | Harvard and Smithsonian. Retrieved from https://asdc.larc.nasa.gov/documents/tempo/guide/TEMPO_Level-2-3_trace_gas_clouds_user_guide_V1.2.pdf

[R24] Heath DF, Krueger AJ, Roeder HA, & Henderson BD (1975). The solar backscatter ultraviolet and total ozone mapping spectrometer (SBUV/TOMS) for Nimbus G. Optical Engineering, 14(4), 323–331. 10.1117/12.7971839

[R25] Heath DF, Mateer CL, & Krueger AJ (1973). The Nimbus-4 Backscatter Ultraviolet (BUV) atmospheric ozone experiment—tow years’ operation. Pure and Applied Geophysics, 106(1), 1238–1253. 10.1007/bf00881076

[R26] Herman J, Cede A, Spinei E, Mount G, Tzortziou M, & Abuhassan N (2009). NO2 column amounts from ground-based Pandora and MFDOAS spectrometers using the direct-sun DOAS technique: Intercomparisons and application to OMI validation. Journal of Geophysical Research, 114(D13). 10.1029/2009jd011848

[R27] Hong H, Kim J, Jeong U, Han KS, & Lee H (2017). The effects of aerosol on the retrieval accuracy of NO2 slant column density. Remote Sensing, 9(8), 867. 10.3390/rs9080867

[R28] Hsu CH, Henze DK, Mizzi AP, González Abad G, He J, Harkins C, (2024). An observing system simulation experiment analysis of how well geostationary satellite trace-gas observations constrain NOx emissions in the US. Journal of Geophysical Research: Atmospheres, 129(2), e2023JD039323. 10.1029/2023jd039323

[R29] Huang Y, Siems ST, Manton MJ, Hande LB, & Haynes JM (2012). The structure of low-altitude clouds over the Southern Ocean as seen by CloudSat. Journal of Climate, 25(7), 2535–2546. 10.1175/jcli-d-11-00131.1

[R30] Judd LM, Al-Saadi JA, Janz SJ, Kowalewski MG, Pierce RB, Szykman JJ, (2019). Evaluating the impact of spatial resolution on tropospheric NO 2 column comparisons within urban areas using high-resolution airborne data. Atmospheric Measurement Techniques, 12(11), 6091–6111. 10.5194/amt-12-6091-2019 33014172 PMC7526561

[R31] Judd LM, Al-Saadi JA, Szykman JJ, Valin LC, Janz SJ, Kowalewski MG, (2020). Evaluating Sentinel-5P TROPOMI tropospheric NO 2 column densities with airborne and Pandora spectrometers near New York City and Long Island Sound. Atmospheric Measurement Techniques, 13(11), 6113–6140. 10.5194/amt-13-6113-2020 34122664 PMC8193800

[R32] Jung J, Choi Y, Mousavinezhad S, Kang D, Park J, Pouyaei A, (2022). Changes in the ozone chemical regime over the contiguous United States inferred by the inversion of NOx and VOC emissions using satellite observation. AtmosphericResearch, 270, 106076. 10.1016/j.atmosres.2022.106076 PMC8972085 35370333

[R33] Kaufman YJ, Tanré D, & Boucher O (2002). A satellite view of aerosols in the climate system. Nature, 419(6903), 215–223. 10.1038/nature01091 12226676

[R34] Keller CA, Knowland KE, Duncan BN, Liu J, Anderson DC, Das S, (2021). Description of the NASA GEOS composition forecast modeling system GEOS-CF v1. 0. Journal of Advances in Modeling Earth Systems, 13(4), e2020MS002413. 10.1029/2020ms002413 PMC8244029 34221240

[R35] Kim HC, Lee P, Judd L, Pan L, & Lefer B (2016). OMI NO2 column densities over North American urban cities: The effect of satellite footprint resolution. Geoscientific Model Development, 9(3), 1111–1123. 10.5194/gmd-9-1111-2016

[R36] Kim J, Jeong U, Ahn MH, Kim JH, Park RJ, Lee H, (2020). New era of air quality monitoring from space: Geostationary Environment Monitoring Spectrometer (GEMS). Bulletin of the American Meteorological Society, 101(1), E1–E22. 10.1175/bams-d-18-0013.1

[R37] Kim S, Kim D, Hong H, Chang LS, Lee H, Kim DR, & Lee K (2023). First-time comparison between NO 2 vertical columns from GEMS and Pandora measurements. Atmospheric Measurement Techniques Discussions, 2023, 1–22.

[R38] Krotkov NA, Lamsal LN, Marchenko SV, Bucsela EJ, Swartz WH, & Joanna & Joiner and the OMI core team. (2019). OMI/Aura nitrogen dioxide (NO2) total and tropospheric column 1-orbit L2 swath 13×24 km V003, Greenbelt, MD, USA [Dataset]. Goddard Earth Sciences Data and Information Services Center (GES DISC). 10.5067/Aura/OMI/DATA2017

[R39] Lambert J-C., Keppens A., Compernolle S., Eichmann K-U., de Graaf M., Hubert D., . (2025). Quarterly validation report of the Copernicus Sentinel-5 Precursor operational data products #26: April 2018 – February 2025 (S5P MPC Routine Operations Consolidated Validation Report Series, Issue #26, Version 26.01.00. Retrieved from https://mpc-vdaf.tropomi.eu/index.php?option=com_vdaf&view=showReport&format=rawhtml&id=64

[R40] Lamsal LN, Krotkov NA, Celarier EA, Swartz WH, Pickering KE, Bucsela EJ, (2014). Evaluation of OMI operational standard NO 2 column retrievals using in situ and surface-based NO 2 observations. Atmospheric Chemistry and Physics, 14(21), 11587–11609. 10.5194/acp-14-11587-2014

[R41] Lamsal LN, Krotkov NA, Vasilkov A, Marchenko S, Qin W, Yang ES, & Bucsela E (2020). OMI/aura nitrogen dioxide standard product with improved surface and cloud treatments. Atmospheric Measurement Techniques Discussions, 2020, 1–56.

[R42] Legates DR, & McCabe GJ Jr (1999). Evaluating the use of “goodness-of-fit” measures in hydrologic and hydroclimatic model validation. Water Resources Research, 35(1), 233–241. 10.1029/1998wr900018

[R43] Levelt PF, Joiner J, Tamminen J, Veefkind JP, Bhartia PK, Stein Zweers DC, (2018). The Ozone Monitoring Instrument: Overview of 14 years in space. Atmospheric Chemistry and Physics, 18(8), 5699–5745. 10.5194/acp-18-5699-2018

[R44] Li KF, Khoury R, Pongetti TJ, Sander SP, Mills FP, & Yung YL (2021). Diurnal variability of stratospheric column NO2 measured using direct solar and lunar spectra over Table Mountain, California (34.38∘ N). Atmospheric Measurement Techniques, 14(12), 7495–7510. 10.5194/amt-14-7495-2021

[R45] Li Y, Xing C, Peng H, Song Y, Zhang C, Xue J, (2023). Long-term observations of NO2 using GEMS in China: Validations and regional transport. Science of the Total Environment, 904, 166762. 10.1016/j.scitotenv.2023.166762 37659571

[R46] Liu G, & Wang Y (2024). Vertical distribution characteristics and potential sources of atmospheric pollutants in the North China Plain basing on the MAX-DOAS measurement. Environmental Sciences Europe, 36(1), 1–14. 10.1186/s12302-024-00902-z

[R47] Liu O, Li Z, Lin Y, Fan C, Zhang Y, Li K, (2024). Evaluation of the first year of Pandora NO 2 measurements over Beijing and application to satellite validation. Atmospheric Measurement Techniques, 17(2), 377–395. 10.5194/amt-17-377-2024

[R48] Logan JA (1983). Nitrogen oxides in the troposphere: Global and regional budgets. Journal of Geophysical Research, 88(C15), 10785–10807. 10.1029/jc088ic15p10785

[R49] Martin RV, Chance K, Jacob DJ, Kurosu TP, Spurr RJ, Bucsela E, (2002). An improved retrieval of tropospheric nitrogen dioxide from GOME. Journal of Geophysical Research, 107(D20), ACH-9. 10.1029/2001jd001027

[R50] Merlaud A, Van Roozendael M, Theys N, Fayt C, Hermans C, Quennehen B, (2011). Airborne DOAS measurements in Arctic: Vertical distributions of aerosol extinction coefficient and NO 2 concentration. Atmospheric Chemistry and Physics Discussions, 11(5), 9219–9236. 10.5194/acp-11-9219-2011

[R51] Munro R, Lang R, Klaes D, Poli G, Retscher C, Lindstrot R, (2016). The GOME-2 instrument on the Metop series of satellites: Instrument design, calibration, and level 1 data processing–an overview. Atmospheric Measurement Techniques, 9(3), 1279–1301. 10.5194/amt-9-1279-2016

[R52] Nouri B, Wilbert S, Segura L, Kuhn P, Hanrieder N, Kazantzidis A, (2019). Determination of cloud transmittance for all sky imager based solar nowcasting. Solar Energy, 181, 251–263. 10.1016/j.solener.2019.02.004

[R53] Nowlan CR, González Abad G, Liu X, Wang H, & Chance K (2025). TEMPO Nitrogen Dioxide Retrieval Algorithm theoretical basis document. NASA Algorithm Publication Tool. 10.5067/WX026254FI2U

[R54] Nowlan CR, Liu X, Janz SJ, Kowalewski MG, Chance K, Follette-Cook MB, (2018). Nitrogen dioxide and formaldehyde measurements from the GEOstationary Coastal and Air Pollution Events (GEO-CAPE) airborne simulator over Houston, Texas. Atmospheric Measurement Techniques, 11(11), 5941–5964. 10.5194/amt-11-5941-2018

[R55] Nowlan CR, Liu X, Leitch JW, Chance K, González Abad G, Liu C, (2016). Nitrogen dioxide observations from the Geostationary Trace gas and Aerosol Sensor Optimization (GeoTASO) airborne instrument: Retrieval algorithm and measurements during DISCOVER-AQ Texas 2013. Atmospheric Measurement Techniques, 9(6), 2647–2668. 10.5194/amt-9-2647-2016

[R56] Oak YJ, Jacob DJ, Pendergrass DC, Dang R, Colombi NK, Chong H, (2025). Air quality trends and regimes in South Korea inferred from 2015–2023 surface and satellite observations. Atmospheric Chemistry and Physics, 25(5), 3233–3252. 10.5194/acp-25-3233-2025

[R57] Palmer PI, Jacob DJ, Chance K, Martin RV, Spurr RJ, Kurosu TP, (2001). Air mass factor formulation for spectroscopic measurements from satellites: Application to formaldehyde retrievals from the Global Ozone Monitoring Experiment. Journal of Geophysical Research, 106(D13), 14539–14550. 10.1029/2000jd900772

[R58] Pandonia Global Network. (2025). Pandora Level-2 TOTNO2 (Direct-Sun total column density of nitrogen dioxide) data from 65 stations used in TEMPO bias analysis, 2023–2024 [Dataset]. NASA Goddard Space Flight Center & European Space Agency. https://data.pandonia-global-network.org/

[R59] Park J, Choi Y, Jung J, Lee K, & Yeganeh AK (2024). First top-down diurnal adjustment to NOx emissions inventory in Asia informed by the Geostationary Environment Monitoring Spectrometer (GEMS) tropospheric NO2 columns. Scientific Reports, 14(1), 24338. 10.1038/s41598-024-76223-1 39420099 PMC11487178

[R60] Pinardi G, Van Roozendael M, Hendrick F, Theys N, Abuhassan N, Bais A, (2020). Validation of tropospheric NO2 column measurements of GOME-2A and OMI using MAX-DOAS and direct sun network observations. Atmospheric Measurement Techniques Discussions, 2020(11), 1–55. 10.5194/amt-13-6141-2020

[R61] Schenkeveld VM, Jaross G, Marchenko S, Haffner D, Kleipool QL, Rozemeijer NC, (2017). In-flight performance of the Ozone Monitoring Instrument. Atmospheric Measurement Techniques, 10(5), 1957–1986. 10.5194/amt-10-1957-2017 29657582 PMC5893161

[R62] Seinfeld JH, & Pandis SN (2016). Atmospheric chemistry and physics: From air pollution to climate change. John Wiley and Sons.

[R63] Szykman J, Swap R, Lefer B, Valin L, Lee SC, Fioletov V, & Robinson J (2019). Pandora: Connecting in-situ and satellite monitoring in support of the Canada–US air quality agreement (pp. 2470–4741). EM: Air and Waste Management Association’s Magazine for Environmental Managers.

[R64] TEMPO_NO2_L3_V03: NASA/LARC/SD/ASDC. (2024). TEMPO gridded NO2 tropospheric and stratospheric columns V03 (PROVISIONAL) [Dataset]. NASA Langley Atmospheric Science Data Center DAAC. 10.5067/IS-40e/TEMPO/NO2_L3.003

[R65] Torres O, Bhartia PK, Jethva H, & Ahn C (2018). Impact of the ozone monitoring instrument row anomaly on the long-term record of aerosol products. Atmospheric Measurement Techniques, 11(5), 2701–2715. 10.5194/amt-11-2701-2018

[R66] Van Donkelaar A, Martin RV, Brauer M, Kahn R, Levy R, Verduzco C, & Villeneuve PJ (2010). Global estimates of ambient fine particulate matter concentrations from satellite-based aerosol optical depth: Development and application. Environmental Health Perspectives, 118(6), 847–855. 10.1289/ehp.0901623 20519161 PMC2898863

[R67] Van Geffen J, Eskes H, Compernolle S, Pinardi G, Verhoelst T, Lambert JC, (2022). Sentinel-5P TROPOMI NO2 retrieval: Impact of version v2.2 improvements and comparisons with OMI and ground-based data. Atmospheric Measurement Techniques, 15(7), 2037–2060. 10.5194/amt-15-2037-2022

[R68] Veefkind JP, Aben I, McMullan K, Förster H, De Vries J, Otter G, (2012). TROPOMI on the ESA Sentinel-5 precursor: A GMES mission for global observations of the atmospheric composition for climate, air quality and ozone layer applications. Remote Sensing of Environment, 120, 70–83. 10.1016/j.rse.2011.09.027

[R69] Verhoelst T, Compernolle S, Pinardi G, Lambert JC, Eskes HJ, Eichmann KU, (2021). Ground-based validation of the Copernicus Sentinel-5p TROPOMI NO 2 measurements with the NDACC ZSL-DOAS, MAX-DOAS and Pandonia global networks. Atmospheric Measurement Techniques, 14(1), 481–510. 10.5194/amt-14-481-2021

[R70] Wang H, Nowlan CR, González Abad G, Chong H, Hou W, Houck JC, (2025). Algorithm theoretical basis for Version 3 TEMPO O2-O2 cloud product. Earth and Space Science, 12(2), e2024EA004165. 10.1029/2024ea004165

[R71] Willmott CJ (1981). On the validation of models. Physical Geography, 2(2), 184–194. 10.1080/02723646.1981.10642213

[R72] Yang LH, Jacob DJ, Colombi NK, Zhai S, Bates KH, Shah V, (2023). Tropospheric NO2 vertical profiles over South Korea and their relation to oxidant chemistry: Implications for geostationary satellite retrievals and the observation of NO2 diurnal variation from space. Atmospheric Chemistry and Physics, 23(4), 2465–2481. 10.5194/acp-23-2465-2023

[R73] Zara M, Boersma KF, De Smedt I, Richter A, Peters E, Van Geffen JH, (2018). Improved slant column density retrieval of nitrogen dioxide and formaldehyde for OMI and GOME-2A from QA4ECV: Intercomparison, uncertainty characterisation, and trends. Atmospheric Measurement Techniques, 11(7), 4033–4058. 10.5194/amt-11-4033-2018

[R74] Zhang C, Liu C, Wang Y, Si F, Zhou H, Zhao M, (2018). Preflight evaluation of the performance of the Chinese environmental trace gas monitoring instrument (EMI) by spectral analyses of nitrogen dioxide. IEEE Transactions on Geoscience and Remote Sensing, 56(6), 3323–3332. 10.1109/tgrs.2018.2798038

[R75] Zoogman P, Liu X, Suleiman RM, Pennington WF, Flittner DE, Al-Saadi JA, (2017). Tropospheric emissions: Monitoring of pollution (TEMPO). Journal of Quantitative Spectroscopy and Radiative Transfer, 186, 17–39. 10.1016/j.jqsrt.2016.05.008 32817995 PMC7430511

