# Supplementary material for: Comprehensive Analysis of Bias in TEMPO NO2 Column Densities Through Pandora Observations
Source: J Geophys Res Atmos. Author manuscript; Available in PMC 2026 Aug 16. (PMC12774391; doi:10.1029/2025JD044150)
Supplement: Supplementary file 1 [file NIHMS2107043-supplement-Supplement1.docx]

*Journal of Geophysical Research: Atmospheres*

Supporting Information for

**Comprehensive Analysis of Bias in TEMPO NO₂ Column Densities through Pandora Observations**

Masoud Ghahremanloo^*1^, Caroline Nowlan^1^, Gonzalo González Abad^1^, Cecilia Garraffo^1^, Xiong Liu^1^, Huiqun Wang^1^, Barron Henderson^2^, Eric Baumann^2^, Lukas Valin^2,^ Jeffrey A. Geddes^3^, Xiaoyi Zhao^4^

1 Center for Astrophysics | Harvard & Smithsonian, Cambridge, MA, USA

2 Center for Environmental Measurements and Modeling, Office of Research and Development, U.S. Environmental Protection Agency, Research Triangle Park, NC, USA

3 Department of Earth & Environment, Boston University, Boston, MA, USA

4 Air Quality Research Division, Environment and Climate Change Canada, Toronto, ON, Canada

Emails: masoud.ghahremanloo@cfa.harvard.edu, [cnowlan@cfa.harvard.edu](mailto:cnowlan@cfa.harvard.edu), [ggonzalezabad@cfa.harvard.edu](mailto:ggonzalezabad@cfa.harvard.edu), [cgarraffo@cfa.harvard.edu](mailto:cgarraffo@cfa.harvard.edu), [xliu@cfa.harvard.edu](mailto:xliu@cfa.harvard.edu), hwang@cfa.harvard.edu, [henderson.barron@epa.gov](mailto:henderson.barron@epa.gov), [baumann.eric@epa.gov](mailto:baumann.eric@epa.gov), valin.lukas@epa.gov, jgeddes@bu.edu, xiaoyi.zhao@ec.gc.ca

*Corresponding author: Masoud Ghahremanloo (masoud.ghahremanloo@cfa.harvard.edu)

**Contents of this file**

Figures S1 to S5

Tables S1 to S2

**Introduction**

Supplementary material for “Comprehensive Analysis of Bias in TEMPO NO₂ Column Densities through Pandora Observations” by M. Ghahremanloo et al. This document contains further figures and tables that help with the interpretation of the results but could not be included in the main manuscript.

**Table S1.** List of Pandonia Global Network (PGN) Pandora stations utilized in this study. The Alt. (Ground) and Alt. (MSL) refer to the altitude of Pandora instrument relative to the ground, and the mean sea level (MSL), respectively.

| Location | Country | Latitude | Longitude | Alt.  (Ground) | Alt.  (MSL) | Start | End |
| --- | --- | --- | --- | --- | --- | --- | --- |
| Aldine, TX | USA | 29.9011 | -95.3262 | 2 | 27 | 05/25/2021 | 12/31/2024 |
| Arlington, TX | USA | 32.7316 | -97.1142 | 10 | 201 | 10/13/2022 | 08/23/2024 |
| Atlanta, GA | USA | 33.7765 | -84.3982 | 20 | 323 | 09/10/2021 | 12/31/2024 |
| Atlanta, GA | USA | 33.7790 | -84.3958 | 31 | 334 | 05/28/2022 | 12/14/2024 |
| Bayonne, NJ | USA | 40.6703 | -74.1261 | 3 | 7 | 05/07/2018 | 12/31/2024 |
| Beltsville, MD | USA | 39.0553 | -76.8783 | 4 | 67 | 06/17/2022 | 12/31/2024 |
| Boston, MA | USA | 42.3497 | -71.1040 | 40 | 52 | 07/03/2019 | 12/26/2024 |
| Boulder, CO | USA | 39.9900 | -105.260 | 1 | 1,736 | 04/30/2018 | 01/25/2024 |
| Boulder, CO | USA | 40.0375 | -105.242 | 14 | 1,632 | 08/26/2021 | 12/31/2024 |
| Bristol, PA | USA | 40.1074 | -74.8824 | 10 | 24 | 03/23/2021 | 12/31/2024 |
| Bronx, NY | USA | 40.8679 | -73.8781 | 12 | 49 | 03/02/2021 | 02/08/2024 |
| Buffalo, NY | Canada | 43.0015 | -78.7869 | 16 | 195 | 05/17/2022 | 12/31/2024 |
| Cambridge, MA | USA | 42.3800 | -71.1100 | 60 | 75 | 01/08/2021 | 11/25/2024 |
| Cameron, LA | USA | 29.7881 | -93.1072 | 2 | 5 | 07/18/2023 | 12/31/2024 |
| Cape Elizabeth, ME | USA | 43.5610 | -70.2073 | 2 | 12 | 04/22/2021 | 10/31/2023 |
| Chapel Hill, NC | USA | 35.9708 | -79.0933 | 50 | 219 | 09/24/2019 | 12/31/2024 |
| Charles City, VA | USA | 37.3260 | -77.2057 | 6 | 16 | 10/18/2019 | 12/07/2024 |
| Chelsea, MA | USA | 42.3946 | -71.0307 | 30 | 40 | 04/24/2024 | 12/31/2024 |
| Chicago, IL | USA | 41.9748 | -87.712 | 10 | 194 | 05/03/2023 | 12/30/2024 |
| Cornwall, CT | USA | 41.8213 | -73.2973 | 7 | 416 | 01/31/2023 | 12/30/2024 |
| Corpus Christi, TX | USA | 27.7150 | -97.3287 | 14 | 17 | 04/05/2024 | 12/31/2024 |
| Dearborn, MI | USA | 42.3067 | -83.1488 | 3 | 184 | 06/09/2021 | 12/31/2024 |
| Downsview | Canada | 43.7810 | -79.4680 | 10 | 202 | 12/03/2017 | 12/12/2024 |
| East Providence, RI | USA | 41.8410 | -71.3610 | 2 | 22 | 04/21/2021 | 03/03/2024 |
| Edwards, CA | USA | 34.9600 | -117.8811 | 2 | 716 | 05/04/2022 | 12/31/2024 |
| Egbert | Canada | 44.2300 | -79.7800 | 2 | 233 | 11/19/2020 | 12/31/2024 |
| Grand Forks, ND | USA | 47.9219 | -97.0865 | 20 | 273 | 05/15/2023 | 12/31/2024 |
| Greenbelt, MD | USA | 38.9926 | -76.8396 | 30 | 85 | 08/24/2021 | 12/03/2024 |
| Hampton, VA | USA | 37.0203 | -76.3366 | 12 | 17 | 02/09/2022 | 11/11/2024 |
| Houston, TX | USA | 29.5790 | -95.2045 | 10 | 24 | 01/09/2024 | 12/22/2024 |
| Houston, TX | USA | 29.7200 | -95.3400 | 8 | 23 | 10/03/2020 | 12/31/2024 |
| Huntsville, AL | USA | 34.7252 | -86.6464 | 11 | 219 | 01/22/2020 | 12/03/2023 |
| Londonderry, NH | USA | 42.8625 | -71.3801 | 4 | 118 | 04/21/2021 | 12/31/2024 |
| Lynn, MA | USA | 42.4746 | -70.9708 | 16 | 38 | 04/24/2024 | 12/31/2024 |
| Madison, CT | USA | 41.2568 | -72.5533 | 2 | 3 | 09/22/2021 | 12/31/2024 |
| Manhattan, NY | USA | 40.8153 | -73.9505 | 32 | 50 | 12/21/2017 | 12/31/2024 |
| Mexico City UNAM | Mexico | 19.3262 | -99.1761 | 13 | 2,286 | 04/20/2018 | 12/31/2024 |
| Mexico City Vallejo | Mexico | 19.4830 | -99.1470 | 9 | 2,252 | 10/24/2019 | 03/29/2024 |
| Mountain View, CA | USA | 37.4200 | -122.0568 | 16 | 24 | 11/21/2019 | 12/31/2024 |
| New Brunswick, NJ | USA | 40.4622 | -74.4294 | 3 | 29 | 09/09/2019 | 03/07/2024 |
| New Haven, CT | USA | 41.3014 | -72.9029 | 4 | 15 | 09/11/2019 | 12/22/2024 |
| New London, CT | USA | 41.3758 | -72.1004 | 20 | 60 | 06/26/2023 | 12/22/2024 |
| Old Field, NY | USA | 40.9635 | -73.1402 | 3 | 6 | 09/12/2019 | 12/31/2024 |
| Philadelphia, PA | USA | 39.9919 | -75.0811 | 6 | 13 | 04/13/2021 | 12/31/2024 |
| Pittsburgh, PA | USA | 40.4655 | -79.9608 | 15 | 278 | 09/02/2021 | 12/31/2024 |
| Queens, NY | USA | 40.7361 | -73.8215 | 10 | 28 | 09/10/2019 | 12/31/2024 |
| Richmond, CA | USA | 37.9130 | -122.336 | 4 | 10 | 06/20/2017 | 12/31/2024 |
| Salt Lake City, UT | USA | 40.7344 | -111.8722 | 4 | 1,324 | 12/01/2022 | 12/31/2024 |
| Salt Lake City, UT | USA | 40.7663 | -111.8478 | 27 | 1,501 | 05/23/2022 | 12/31/2024 |
| San Jose, CA | USA | 37.3325 | -121.8821 | 30 | 56 | 11/01/2020 | 12/31/2024 |
| South Jordan, UT | USA | 40.5480 | -112.070 | 2 | 1,595 | 05/26/2022 | 08/12/2024 |
| St George | Canada | 43.6605 | -79.3986 | 61 | 180 | 05/08/2023 | 12/31/2024 |
| SW Detroit, MI | USA | 42.3026 | -83.1068 | 2 | 183 | 06/09/2021 | 04/24/2024 |
| Toronto CN Tower | Canada | 43.6426 | -79.3871 | 330 | 426 | 02/08/2024 | 12/31/2024 |
| Toronto Scarborough | Canada | 43.7843 | -79.1874 | 4 | 137 | 12/18/2019 | 12/22/2024 |
| Toronto West | Canada | 43.7094 | -79.5435 | 7 | 155 | 11/05/2021 | 12/31/2024 |
| Tucson, AZ | USA | 32.2330 | -110.953 | 11 | 752 | 12/07/2023 | 12/25/2024 |
| Turlock, CA | USA | 37.5230 | -120.855 | 13 | 47 | 05/31/2023 | 12/31/2024 |
| Tyler, TX | USA | 32.3440 | -95.4159 | 1 | 160 | 09/24/2024 | 12/31/2024 |
| Virginia Beach, VA | USA | 37.0367 | -76.0766 | 8 | 8 | 03/14/2024 | 12/10/2024 |
| Washington DC | USA | 38.9218 | -77.0124 | 4 | 61 | 08/06/2020 | 12/09/2024 |
| Westport, CT | USA | 41.1183 | -73.3367 | 1 | 6 | 04/20/2021 | 10/03/2024 |
| Whittier, CA | USA | 33.9768 | -118.0299 | 23 | 131 | 05/08/2023 | 12/31/2024 |
| Windsor West | Canada | 42.2929 | -83.0731 | 3 | 184 | 07/13/2022 | 12/31/2024 |
| Wright Wood, CA | USA | 34.3819 | -117.6813 | 3 | 2,036 | 11/20/2019 | 12/31/2024 |

**Figure S1.** Performance of TEMPO TOTNO_2_ across different cloud cover ranges: (a) histogram of cloud fraction ranges for TEMPO-Pandora pairs, (b) correlation coefficient (R) and index of agreement (IOA), (c) percentage bias metrics (MABP, MBP, RMSEP), and (d) absolute bias metrics (MAB, MB, RMSE).

**Table S2.** Accuracy of TEMPO TOTNO_2_ in various Pandora stations in the TEMPO domain. The validation metrics are the correlation coefficient (R), the index of agreement (IOA), the mean bias (MB: TEMPO minus Pandora), the mean absolute bias (MAB), and the root mean square error (RMSE). The MAB, MB, and RMSE are reported in ×10^15^ molecules/cm^2^ and % refers to the percentage bias, which is MAB, MB, or RMSE divided by mean TOTNO_2_ in Pandora station. Altitude (Alt.) refers to the height of the Pandora instrument relative to the ground, while Observations (Obs.) indicate the number of TEMPO-Pandora pairs at each station. The stations are sorted in descending order based on their IOA values.

| Location | Lat. | Long. | Alt. | Obs. | R | IOA | MAB (%) | MB (%) | RMSE (%) |
| --- | --- | --- | --- | --- | --- | --- | --- | --- | --- |
| Bronx, NY | 40.8679 | -73.8781 | 12 | 296 | 0.84 | 0.90 | 3.72 (32.2) | 1.78 (15.4) | 5.75 (49.7) |
| Westport, CT | 41.1183 | -73.3367 | 1 | 1069 | 0.80 | 0.89 | 1.51 (23.1) | 0.50 (7.7) | 2.24 (34.3) |
| Old Field, NY | 40.9635 | -73.1402 | 3 | 1739 | 0.78 | 0.88 | 1.33 (19.6) | 0.01 (0.2) | 1.90 (28) |
| Salt Lake City, UT | 40.7344 | -111.8722 | 4 | 1754 | 0.79 | 0.88 | 1.92 (24.1) | 0.49 (6.2) | 2.59 (32.5) |
| Chicago, IL | 41.9748 | -87.7120 | 10 | 1403 | 0.77 | 0.88 | 2.04 (20.2) | -0.017 (-0.2) | 3.18 (31.5) |
| Madison, CT | 41.2568 | -72.5533 | 2 | 1457 | 0.77 | 0.86 | 0.93 (18.1) | 0.159 (3.1) | 1.23 (23.9) |
| Houston, TX | 29.7200 | -95.3400 | 8 | 1500 | 0.79 | 0.86 | 2.89 (23.4) | -1.71 (-13.9) | 4.37 (35.4) |
| South Jordan, UT | 40.5480 | -112.070 | 2 | 740 | 0.77 | 0.85 | 1.35 (21.4) | -0.373 (-5.9) | 2.15 (34) |
| Aldine, TX | 29.9011 | -95.3262 | 2 | 1631 | 0.73 | 0.85 | 2.12 (22.9) | -0.010 (-0.1) | 3.08 (33.3) |
| New Brunswick, NJ | 40.4622 | -74.4294 | 3 | 461 | 0.75 | 0.85 | 2.65 (27.6) | 0.811 (8.4) | 4.39 (45.7) |
| Scarborough, ON | 43.7843 | -79.1874 | 4 | 899 | 0.72 | 0.83 | 2.13 (24.4) | -0.54 (-6.2) | 3.11 (35.7) |
| Arlington, TX | 32.7316 | -97.1142 | 10 | 872 | 0.70 | 0.83 | 1.30 (17.9) | 0.001 (0) | 1.88 (25.8) |
| Toronto West, ON | 43.7094 | -79.5435 | 7 | 749 | 0.71 | 0.82 | 2.72 (23.7) | -1.16 (-10.1) | 4.07 (35.5) |
| Downsview, ON | 43.7810 | -79.4680 | 10 | 2568 | 0.71 | 0.82 | 2.28 (26.8) | 0.9 (10.6) | 3.53 (41.5) |
| Salt Lake City, UT | 40.7663 | -111.8478 | 27 | 789 | 0.69 | 0.82 | 2.17 (25.6) | -0.457 (-5.4) | 3.09 (36.5) |
| Houston, TX | 29.5790 | -95.2045 | 10 | 1042 | 0.78 | 0.82 | 2.47 (27.9) | -1.99 (-22.5) | 3.18 (35.9) |
| San Jose, CA | 37.3325 | -121.8821 | 30 | 2371 | 0.68 | 0.82 | 1.66 (19.6) | -0.454 (-5.3) | 2.40 (28.3) |
| Bayonne, NJ | 40.6703 | -74.1261 | 3 | 1420 | 0.71 | 0.81 | 4.88 (27.1) | -2.62 (-14.5) | 6.96 (38.6) |
| Boulder, CO | 40.0375 | -105.242 | 14 | 1505 | 0.77 | 0.81 | 1.10 (19.7) | -0.82 (-14.7) | 1.57 (28) |
| Tucson, AZ | 32.2330 | -110.953 | 11 | 1944 | 0.66 | 0.81 | 1.12 (18) | -0.056 (-0.9) | 1.56 (25) |
| Cameron, LA | 29.7881 | -93.1072 | 2 | 1697 | 0.67 | 0.80 | 0.66 (19.1) | 0.15 (4.4) | 0.925 (27) |
| Manhattan, NY | 40.8153 | -73.9505 | 32 | 1223 | 0.72 | 0.80 | 5.71 (48.7) | 4.17 (35.6) | 8.21 (70.1) |
| Corpus Christi, TX | 27.7150 | -97.3287 | 14 | 744 | 0.71 | 0.80 | 0.751 (19) | -0.41 (-10.2) | 1.02 (25.7) |
| Grand Forks, ND | 47.9219 | -97.0865 | 20 | 864 | 0.67 | 0.79 | 0.709 (15) | -0.349 (-7.4) | 0.91 (19.2) |
| Queens, NY | 40.7361 | -73.8215 | 10 | 1265 | 0.65 | 0.79 | 4.62 (30.5) | 0.007 (0.1) | 7.40 (48.8) |
| Egbert, ON | 44.2300 | -79.7800 | 2 | 880 | 0.67 | 0.79 | 0.879 (21) | 0.369 (8.8) | 1.40 (33.3) |
| New Haven, CT | 41.3014 | -72.9029 | 4 | 1501 | 0.69 | 0.78 | 2.26 (27.4) | -0.661 (-8) | 3.27 (39.6) |
| Cornwall, CT | 41.8213 | -73.2973 | 7 | 1024 | 0.67 | 0.78 | 0.97 (22.6) | 0.255 (5.9) | 1.46 (33.8) |
| Mexico City Vallejo | 19.4830 | -99.1470 | 9 | 868 | 0.79 | 0.78 | 10 (31.5) | -8.5 (-26.7) | 13.1 (41.1) |
| Richmond, CA | 37.9130 | -122.336 | 4 | 2249 | 0.64 | 0.77 | 1.38 (24.7) | 0.67 (12) | 1.85 (33.2) |
| Philadelphia, PA | 39.9919 | -75.0811 | 6 | 1277 | 0.67 | 0.77 | 2.85 (29) | 0.834 (8.5) | 4.57 (46.4) |
| Mexico City UNAM | 19.3262 | -99.1761 | 13 | 2112 | 0.62 | 0.77 | 7.43 (29.9) | -2.51 (-10.1) | 10.4 (42.1) |
| East Providence, RI | 41.8410 | -71.3610 | 2 | 399 | 0.65 | 0.77 | 1.55 (23.8) | 0.354 (5.4) | 2.36 (36.3) |
| Windsor West, ON | 42.2929 | -83.0731 | 3 | 1041 | 0.62 | 0.76 | 2.69 (29.5) | -0.136 (-1.5) | 3.96 (43.3) |
| Virginia Beach, VA | 37.0367 | -76.0766 | 8 | 606 | 0.61 | 0.76 | 1.0 (21.4) | 0.181 (3.9) | 1.29 (27.6) |
| Bristol, PA | 40.1074 | -74.8824 | 10 | 1432 | 0.71 | 0.75 | 2.74 (34.4) | 1.92 (24.1) | 4.35 (54.4) |
| Pittsburgh, PA | 40.4655 | -79.9608 | 15 | 1205 | 0.68 | 0.75 | 2.2 (29.72) | 1.19 (16.1) | 3.59 (48.4) |
| Dearborn, MI | 42.3067 | -83.1488 | 3 | 1095 | 0.59 | 0.74 | 3.30 (29.1) | -1.34 (-11.8) | 4.85 (42.8) |
| Atlanta, GA | 33.7765 | -84.3982 | 20 | 1528 | 0.59 | 0.74 | 2.22 (26.3) | 0.536 (6.4) | 3.21 (38.1) |
| Beltsville, MD | 39.0553 | -76.8783 | 4 | 503 | 0.72 | 0.74 | 2.88 (38.5) | 2.22 (29.7) | 4.38 (58.6) |
| Washington DC | 38.9218 | -77.0124 | 4 | 1403 | 0.63 | 0.74 | 2.47 (27.4) | 0.648 (7.2) | 3.96 (43.9) |
| Edwards, CA | 34.9600 | -117.8811 | 2 | 1971 | 0.65 | 0.73 | 0.711 (17) | 0.356 (8.3) | 0.96 (22.4) |
| SW Detroit, MI | 42.3026 | -83.1068 | 2 | 281 | 0.59 | 0.73 | 3.6 (29.8) | -2.02 (-16.8) | 5.07 (42) |
| Whittier, CA | 33.9768 | -118.0299 | 23 | 2274 | 0.58 | 0.73 | 4.36 (35.9) | 2.24 (18.5) | 6.08 (50.1) |
| St George, Toronto | 43.6605 | -79.3986 | 61 | 597 | 0.56 | 0.72 | 2.74 (28.6) | 0.581 (6.1) | 4.34 (45.3) |
| Wright wood, CA | 34.3819 | -117.6813 | 3 | 2247 | 0.61 | 0.71 | 0.711 (20) | 0.42 (11.9) | 1.09 (30.6) |
| Hampton, VA | 37.0203 | -76.3366 | 12 | 1602 | 0.55 | 0.71 | 1.32 (22) | -0.117 (-2) | 1.87 (31.2) |
| Greenbelt, MD | 38.9926 | -76.8396 | 30 | 2838 | 0.65 | 0.70 | 2.30 (33.8) | 1.64 (24.1) | 3.47 (50.9) |
| Londonderry, NH | 42.8625 | -71.3801 | 4 | 1267 | 0.66 | 0.70 | 1.16 (27.5) | 0.74 (17.7) | 1.71 (40.8) |
| Mountain View, CA | 37.4200 | -122.0568 | 16 | 2264 | 0.65 | 0.69 | 1.47 (26.6) | 1.19 (21.5) | 2.02 (36.5) |
| Chelsea, MA | 42.3946 | -71.0307 | 30 | 593 | 0.52 | 0.68 | 2.18 (29.4) | -0.111 (-1.5) | 3.39 (45.6) |
| Boulder, CO | 39.9900 | -105.260 | 1 | 362 | 0.75 | 0.66 | 2.10 (30.3) | -2.03 (-29.2) | 2.73 (39.3) |
| Tyler, TX | 32.3440 | -95.4159 | 1 | 403 | 0.49 | 0.66 | 0.780 (18) | -0.008 (-1.9) | 1.05 (24.3) |
| Huntsville, AL | 34.7252 | -86.6464 | 11 | 378 | 0.48 | 0.66 | 1.02 (19) | -0.29 (-5.4) | 1.31 (24.5) |
| Lynn, MA | 42.4746 | -70.9708 | 16 | 787 | 0.53 | 0.65 | 1.93 (33.3) | 1.14 (19.6) | 2.96 (51.0) |
| Buffalo, NY | 43.0015 | -78.7869 | 16 | 965 | 0.51 | 0.63 | 1.31 (25) | 0.80 (15.3) | 1.88 (35.8) |
| Cambridge, MA | 42.3800 | -71.1100 | 60 | 538 | 0.42 | 0.62 | 2.14 (32.1) | 0.92 (13.7) | 3.17 (47.5) |
| Atlanta, GA | 33.7790 | -84.3958 | 31 | 1594 | 0.39 | 0.61 | 2.72 (30.4) | -0.002 (-0.3) | 4.11 (46) |
| New London, CT | 41.3758 | -72.1004 | 20 | 448 | 0.55 | 0.59 | 1.71 (38.7) | 1.32 (28.9) | 2.72 (59.6) |
| Turlock, CA | 37.5230 | -120.855 | 13 | 2538 | 0.31 | 0.57 | 1.11 (19.1) | -0.159 (-2.7) | 1.49 (25.5) |
| Charles City, VA | 37.3260 | -77.2057 | 6 | 1031 | 0.46 | 0.56 | 1.89 (29.1) | -1.35 (-20.9) | 2.99 (46.2) |
| Chapel Hill, NC | 35.9708 | -79.0933 | 50 | 1499 | 0.46 | 0.56 | 1.32 (30.7) | 0.84 (19.6) | 1.82 (42.3) |
| Boston, MA | 42.3497 | -71.104 | 40 | 556 | 0.29 | 0.55 | 2.32 (28.8) | -0.336 (-4.2) | 3.47 (43) |
| Cape Elizabeth, ME | 43.5610 | -70.2073 | 2 | 171 | 0.40 | 0.52 | 0.784 (20) | 0.295 (7.5) | 1.16 (29.6) |
| CN Tower, Toronto | 43.6426 | -79.3871 | 330 | 618 | 0.36 | 0.51 | 3.19 (49.3) | 2.82 (43.5) | 4.60 (71.1) |

**Figure S2.** Performance of TEMPO TOTNO_2_ in various Pandora stations in the TEPMO domain. The map shows the values of the correlation coefficient (R) between TEMPO TOTNO_2_ and Pandora observations.

**Figure S3.** Spatial distribution of TEMPO TOTNO_2_ performance across Pandora stations within the TEMPO domain. The map illustrates the percentage mean absolute bias (MABP) of TEMPO TOTNO_2_ at various Pandora stations. MABP is calculated as MAB normalized by the mean Pandora TOTNO_2_.

**Figure S4.** Spatial distribution of TEMPO TOTNO_2_ performance across Pandora stations within the TEMPO domain. The map illustrates the percentage mean bias (MBP) of TEMPO TOTNO_2_ at various Pandora stations. MBP is calculated as MB (TEMPO TOTNO_2_ minus Pandora observations), normalized by the mean Pandora observation.

**Figure S5.** Spatial distribution of TEMPO TOTNO_2_ performance across Pandora stations within the TEMPO domain. The map illustrates the percentage root mean square error (RMSEP) of TEMPO TOTNO_2_ at various Pandora stations. RMSEP is calculated as RMSE normalized by the mean Pandora TOTNO_2_.
